# 3,6′-Disinapoyl Sucrose from *Polygalae Radix* Exerts Anti-Aging Effects via Modification of Telomeres, SIRT1/p53/p21 Pathway, Oxidative Stress and Autophagy

**DOI:** 10.3390/antiox15030313

**Published:** 2026-03-01

**Authors:** Jianhong Wang, Ting Jiang, Siqi Chen, Yajing Li, Qing Li, Lan Xiang, Jianhua Qi

**Affiliations:** 1 College of Chemistry and Materials Science, Sichuan Normal University, Chengdu 610068, China; 20231201025@stu.sicnu.edu.cn (J.W.); 20221201040@stu.sicnu.edu.cn (S.C.); 2 College of Pharmaceutical Sciences, Zhejiang University, Yu Hang Tang Road 866, Hangzhou 310058, China; 22319087@zju.edu.cn (T.J.); 12019045@zju.edu.cn (Y.L.); qijianhua@zju.edu.cn (J.Q.)

**Keywords:** *Polygalae Radix*, 3,6′-disinapoyl sucrose, SIRT1, telomere, autophagy, anti-oxidative stress

## Abstract

Traditional Chinese medicine plays an important role in human health, but due to the complexity of its active fraction, its therapeutic mechanism still needs further clarification. *Polygalae Radix* is one of the traditional Chinese medicines, which was recorded in *Shennong Classic of Materia Medica* with the effect of prolonging life. In the present study, we isolated a small molecule compound with anti-aging effects, 3,6′-disinapoyl sucrose (DISS), from *Polygalae Radix* under the guidance of the replicative lifespan assay of K6001 yeast strain. It extended the lifespan of yeast and alleviated etoposide-induced aging in 3T3 cells. Furthermore, this compound increased the telomerase activity and the length of telomeres, and targeted the SIRT1 signaling pathway, respectively. In addition, it improved the survival ability of yeast under oxidative stress conditions, decreased ROS and MDA levels, and increased the activity of SOD, CAT and GPx enzymes. Moreover, DISS enhanced autophagic flux, as demonstrated by assay of the YOM38-GFP-ATG8 yeast strain. In conclusion, DISS from *Polygalae Radix* exerts anti-aging effects by protecting telomeres, regulating the SIRT1/p53/p21 signaling pathway, mitigating oxidative stress and modulating autophagy. Thus, this study provides scientific evidence for the use of *Polygalae Radix* as an anti-aging herb.

## 1. Introduction

The global elderly population is growing at an unprecedented rate, with those aged 65 and above reaching 800 million in 2024, accounting for approximately 10% of the world’s total population [1]. At the same time, the probability of the elderly suffering from a variety of comorbidities increases with age, including Alzheimer’s disease (AD), diabetes, osteoporosis (OP), cancer and other diseases [2]. Although numerous medications can alleviate the diseases associated with aging, achieving effective treatment requires different combinations of treatment methods, long-term treatment, and a large amount of funding, which imposes a huge burden on families and society [3]. Therefore, we hypothesize that if we can prevent aging, we might be able to reduce the occurrence of these diseases. To achieve this goal, the discovery of small molecules with anti-aging effects and clarifying their mechanism of action are very important.

Silent information regulator 2 (Sir2) is a type III histone deacetylase that relies on nicotinamide adenine dinucleotide (NAD+) [4]. SIRT1 in mammals is homologous to Sir2 and plays a central role in maintaining genomic stability by participating in chromatin remodeling, regulating DNA damage response, and interacting with repair factors [4]. Meanwhile, SIRT1 is also implicated in the regulation of telomeres [4]. Telomeres primarily maintain genomic stability, regulate cell proliferation and aging [2]. Before cells enter senescence, telomeres can be recognized and bound by shelterin complexes to protect their structures and to avoid being mistaken for DNA damage, thereby delaying cellular aging [5]. At the same time, telomerase can directly compensate for lost end DNA sequences by increasing telomere repeat sequences, which also provides a binding platform for telomere-specific binding proteins such as shelterin complexes [6].

Besides the inherent replication defects at the ends of chromosomes, telomere shortening is also accelerated by oxidative stress that preferentially attacks telomere DNA. Oxidative stress refers to an imbalance in which the production of reactive oxygen species (ROS) in the body exceeds the defense capacity of the endogenous antioxidant system, leading to a disruption of the oxidative-antioxidant balance and a tilt towards the oxidative environment. This state can cause damage to biomolecules (proteins, lipids, DNA) and is closely related to physiological and pathological processes such as inflammation and aging [7]. Specific organelles within cells, such as mitochondria, produce ROS through normal metabolism, related enzymatic reactions, and exogenous stimuli. ROS can cause lipid peroxidation chain reactions, producing unstable intermediate products such as lipid peroxidation (LOOH). LOOH will further decompose into various toxic substances, such as malondialdehyde (MDA) and 4-hydroxynonenal (4-HNE). These compounds can damage biomolecules, exacerbate oxidative stress, disrupt cellular signaling pathways, and accelerate cellular senescence and organismal aging [8]. Common endogenous enzyme antioxidants, including superoxide dismutase (SOD), catalase (CAT), and glutathione peroxidase (GPx), work together to metabolize ROS into non-toxic molecules [9]. Meanwhile, SIRT1 can inhibit the activity of transcription factor p53 through deacetylation and reduce the expression of pro-oxidative genes, thereby suppressing the production of ROS and MDA and increasing the activity of antioxidant enzymes [10].

Excessive ROS levels also directly act as damage signals, activating cellular clearance processes including mitochondrial autophagy [11]. Autophagy is a self-cleaning process in which cells package and transport erroneous or damaged components to lysosomes for degradation or recycling through three pathways: microautophagy, chaperone-mediated autophagy, and macroautophagy. This process maintains cellular protein homeostasis, reduces the accumulation of cellular damage, and delays aging [12]. Some studies have indicated that regulating ATG protein can enhance autophagy, thereby effectively delaying aging and extending healthy lifespan [11,13]. So far, the 41 *ATG* genes that mediate the formation of autophagosomes in yeast have been identified, including Atg2 protein, which is involved in lipid transport, and Atg32 protein, which is a specific receptor for mitochondrial autophagy, encoded by the *ATG2* and *ATG32* genes, respectively [11,13]. The Atg32 protein works by binding to the autophagosome marker proteins Atg8 and Atg11 [11]. Meanwhile, SIRT1 can participate in autophagy by deacetylating autophagic ATG proteins (ATG5, ATG7, ATG8), degrading organelles and proteins [7].

*Saccharomyces cerevisiae* is an advantageous model for aging research due to its clear genetic background, convenient genetic operation, short life cycle, and highly conservative aging regulating mechanism [11]. As the first eukaryotic organism to be fully sequenced, yeast shares a large number of homologous genes with humans [14]. During aging research, the replicative lifespan (RLS) and chronological lifespan (CLS) of yeast were measured [15]. RLS and CLS refer to the limit of the number of divisions a cell can undergo under in vitro culture conditions and to the length of time a cell can survive and function after it has stopped dividing, respectively. They are often used to measure the cellular senescence process. In order to achieve efficient, quantitative, and comparable mechanism exploration, the K6001 yeast strain was used for the replicative lifespan assay in this study [16].

*Polygalae Radix* is one of the traditional Chinese medicines. According to the *Shennong Classic of Materia Medica*, it is beneficial for wisdom, improves hearing and vision, long-lasting consumption, prolongs life and healthily preserves vitality [17]. This medicine mainly contains chemical components such as triterpenoid saponins, flavonoids, oligosaccharide esters, and alkaloids. Some evidence suggests that these molecules possess sedative, hypnotic, antidepressant, anti-aging, and anti-inflammatory activity [17,18]. In the present study, we performed the separation of *Polygalae Radix* under the guidance of the K6001 yeast replicative lifespan assay. The 3,6′-disinapoyl sucrose (DISS) with novel anti-aging effects was found. To deeply understand the mechanism of action for this compound, we focused on the identification of the target protein and the mechanism of action of DISS to do research in this study. Here, we reported that DISS exerted anti-aging effects via targeting and activating the SIRT1 protein and modulating telomere, oxidative stress, and autophagy.

## 2. Materials and Methods

### 2.1. General and Yeast Strains

The dichloromethane (DCM), ethyl acetate (EA), ethanol (EtOH), and methanol (MeOH) were supplied by Sinopharm Chemical Reagent Co., Ltd. (Shanghai, China). Dimethyl sulfoxide (DMSO) and deuterated methanol (CD_3_OD) were purchased from Shanghai Aladdin Biochemical Technology Co., Ltd. (Shanghai, China) and J&K Scientific Ltd. (Beijing, China), respectively. Silica gel (200–300 mesh) and reversed-phase C18 (Octadecylsilyl, ODS) were obtained from the Yantai Research Institute of Chemical Industry (Yantai, China) and Nacalai Tesque, Inc. (Kyoto, Japan), respectively. ^1^H NMR spectra data were recorded on a Bruker AV III-500 spectrometer (Bruker, Karlsruhe, Germany). The compounds, such as resveratrol (RES), rapamycin (Rapa), and astragaloside IV (AST) were sourced from J&K Scientific Ltd. (Beijing, China), Solarbio (Beijing, China), and Yuanye Bio-Technology Co., Ltd. (Shanghai, China), respectively. In this study, several positive controls were used in different models, and the details of doses, compounds, selected reasons, and references are described in Supplementary Table S1. EtOH and DMSO were used as solvents to dissolve the compounds or as a negative control for yeast-related or NIH/3T3 cells experiments, respectively. The common media and reagent configurations are described in Supplementary Information Section S1.1.

In this study, the K6001 yeast was donated by Prof. Michael Breitenbach from the University of Salzburg, Austria. The following yeast strains were gifted by Prof. Akira Matsuura from Chiba University, Japan: BY4741; K6001 background mutants (*Δsod1*, *Δsod2*, *Δcat*, *Δgpx*, *Δatg2*, *Δatg32*) and YOM38 plasmid containing pR316-GFP-ATG8. The S288C yeast strain was purchased from Hangzhou Baosai Biotechnology Co., Ltd. (Hangzhou, China). The genotypes of all yeast strains mentioned above are listed in the Supplementary Table S2. Mouse embryonic fibroblasts (NIH/3T3) were purchased from Meilun Biotechnology Co., Ltd., (Dalian, China).

The first antibodies used in this experiment were as follows: SIRT1 antibody (Cell Signaling Technology, Shanghai, China, #9475, 1:1000), GFP antibody (Wuhan Sanying Biotechnology Co., Ltd., Wuhan, China, 50430-2-AP, 1:1000), p53 antibody (Hangzhou Huaan Biotechnology Co., Ltd., Hangzhou, China, HA722074, 1:1000), p21 antibody (Hangzhou Huaan Biotechnology Co., Ltd., Hangzhou, China, HA722065, 1:1000), TRF2 antibody (Cell Signaling Technology, Shanghai, China, #13136, 1:1000), and RAP1 antibody (Cell Signaling Technology, Shanghai, China, #5433, 1:1000), and *β*-actin antibody (Hangzhou Daige Biotech Co., Ltd., Hangzhou, China, #db13986, 1:1000). The second antibodies used were Goat Anti-Rabbit IgG, HRP Conjugated (Jiangsu Kangwei Century Biotechnology Co., Ltd., Nanjing, China, CW0103S, 1:5000) and Goat Anti-Mouse IgG, HRP Conjugated (Jiangsu Kangwei Century Biotechnology Co., Ltd., Nanjing, China, CW0102S, 1:5000).

### 2.2. Isolation and Purification of DISS

**Figure 1:**
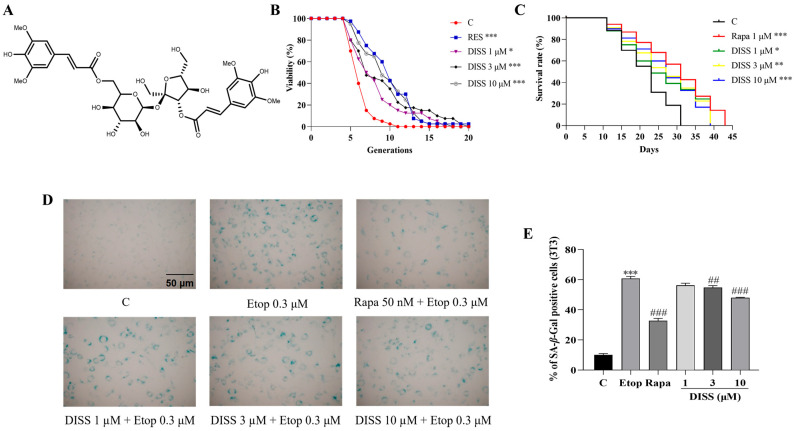
The chemical structure of DISS and the anti-aging effects of DISS on yeast and NIH/3T3 cells. (**A**) The chemical structure of DISS. (**B**) Effects of 10 μM RES or 0, 1, 3, 10 μM DISS on the replicative lifespan of K6001 yeast. (**C**) Effects of 1 μM Rapa or 0, 1, 3, 10 µM DISS on the chronological lifespan of BY4741 yeast. (**D**) Anti-aging capability of DISS and Rapa in Etop-induced NIH/3T3 senescent cells. (**E**) The digital result of D. For the RLS, viability was measured using three biological replicates. (*n* = 3). For the CLS timepoints, the survival rate was measured using three technical replicates (*n* = 3). *, ** and *** represent significant differences at *p* < 0.05, *p* < 0.01, and *p* < 0.001 compared with negative control group, respectively. ^##^ and ^###^ represent significant differences at *p* < 0.01 and *p* < 0.001 compared with the Etop group, respectively.

*Polygalae Radix* was purchased from Yili Pharmaceutical Co., Ltd. (Bozhou, China). The *Polygalae Radix* powder (50 g) was ultrasonically extracted with 10 times the amount of methanol at room temperature for 30 min. This process was repeated three times. The residue was further extracted three times with ethyl acetate. Then, the combined supernatants were concentrated under vacuum to obtain 19.1 g of crude extract. The extract of this medicine was subjected to silica open column and eluted with EA/MeOH (90:10, 70:30, 50:50, 30:70 and 0:100) to obtain six fractions from II-A-1 to II-A-6 (mass yield: 2.4%, 18.1%,11.9%, 19.2%, 20.3%, 23.1%). All of the fractions were done in the replicative lifespan assay, and the fraction that extended the longest replicative lifespan of yeast was selected. The most active fraction II-A-3 (2.28 g) was separated on silica open column with DCM/MeOH (100:0; 95:5; 90:10; 85:5; 80:20; 75:25; 70:30; 50:50 and 0:100) to obtain six fractions from II-B-1 to II-B-6 (mass yield: 2.6%, 4.9%, 3.1%, 12.2%, 23.1%, 38.8%). After activity measurement, the active fraction II-B-3 (61.6 mg) was separated again using ODS (Octadecylsilyl) open column with CH_3_OH/H_2_O (10:90, 20:80, 30:70, 40:60, 50:50 and 100:0) to obtain six fractions from II-C-1 to II-C-6 (mass yield:14.5%, 11.2%, 19.8%, 16.3%, 12.7%, 5.5%) and took replicative lifespan assay. The most active II-C-2 fraction (6.7 mg) was confirmed by thin-layer chromatography (TLC) analysis using both normal-phase and reversed-phase plates to check purification. After that, the chemical structure of the II-C-2 fraction with novel anti-aging effects was determined with ^1^H NMR analysis and compared with the literature [19]. ^1^H-NMR (500 MHz, CD_3_OD, δ) 7.69 (^1^H, d, J = 15.8 Hz), 7.61 (^1^H, d, J = 15.9 Hz), 6.95 (2H, s), 6.90 (2H, s), 6.49 (^1^H, d, J = 7.3 Hz), 6.46 (^1^H, d, J = 7.3Hz), 5.53 (2H, m), 4.70 (^1^H, d, J = 11.6 Hz), 4.52 (^1^H, t, J = 8.1 Hz), 4.30 (^1^H, m), 4.22 (^1^H, dd, J = 11.7, 7.3 Hz),3.98 (^1^H, dd, J = 7.9, 3.1 Hz), 3.92 (^1^H, m), 3.85 (^1^H, d, J = 5.0 Hz), 3.82 (^1^H, m), 3.68 (2H, t, J = 9.3 Hz), 3.61 (^1^H, m), 3.49 (^1^H, dd, J = 9.7, 3.8 Hz), 3.89 (6H, s), 3.86 (6H, s). The chemical structure of this compound and the ^1^H-NMR spectrum are displayed in Figure 1A and Supplementary Figure S1, respectively.

### 2.3. Replicative and Chronological Lifespan Assay

The replicative lifespan assay was performed according to the previous report [20]. Briefly, the K6001 yeast stored at −30 °C was washed three times with phosphate-buffered saline (PBS), resuspended in galactose medium, and incubated in a 180 rpm shaker at 28 °C for 24 h. After that, 1 mL of K6001 yeast culture was transferred to a 15 mL centrifuge tube and centrifuged to pellet the cells. The cells were washed three times with PBS and counted with a hemocytometer. The 40 μL of suspension containing approximately 4000 yeast cells was smeared on the yeast peptone dextrose (YPD) agar plates containing 0, 1, 3, 10 μM DISS or 10 μM RES, respectively. Subsequently, these plates were incubated at 28 °C for 48 h, and 40 microcolonies formed on the agar plate of each group were randomly selected to count the offspring produced by the metrocytes through observation with an upright microscope (Olympus Corporation, Tokyo, Japan). To avoid failed growth colonies and overlapping colonies, the microcolonies with the number of daughter cells less than 3 or more than 20 were not counted during the replicative lifespan assay. RES is a famous molecule with anti-aging effects on a wide range of organisms, so we used RES as a positive control to demonstrate the reliability of the experimental results, and ethanol was used to dissolve the samples and served as a negative control. In addition, the replicative lifespan assay of yeast mutant strains with a K6001 background (*Δsod1*, *Δsod2*, *Δcat*, *Δgpx*, *Δatg2*, *Δatg32*) was similar to that of K6001 yeast.

The anti-aging ability of BY4741 yeast with 1, 3, 10 μM DISS was tested according to the previous study [20]. Briefly, the BY4741 yeast strain stored at −30 °C was washed three times with PBS, resuspended in 5 mL of glucose liquid medium, and cultured in a 180 rpm shaker at 28 °C for 24 h. Afterwards, the yeast cell suspension was inoculated in a new 100 mL SC medium with an initial OD_600_ value of 0.01 in a 250 mL flask, treated with DISS at concentrations of 0, 1, 3, 10 μM or 1 μM Rapa, and incubated in a shaker at 28 °C. On the third day of culture, 100 μL of culture medium was taken to count the cells with a hemacytometer, and nearly 200 yeast cells of each group were spread on the glucose medium agar plate and repeated three times, respectively. After these plates were incubated at 28 °C for 48 h, the number of colony-forming units (CFUs) of each group was counted. This process was repeated every 4 days, and an equal volume of SC medium without yeast was added to the flasks until the colony viability of each group was less than 10%. The CFUs at day 3 were fixed as a 100% survival. (The survival rate = CFUs/CFUs on day 3 × 100%).

### 2.4. Senescence-Associated β-Galactosidase (SA–β-Gal) Staining Assay in Etoposide-Induced Aging Cells

In this experiment, 3T3 cells were first seeded to achieve an initial density of 5 × 10^4^ cells per well in a 24-well plate. Then, the cells were cultured at 37 °C with 5% CO_2_ for 24 h. Subsequently, these cells were treated with DISS at doses of 1, 3, 10 μM, 0.5% DMSO or 50 nM Rapa for 24 h, respectively. To induce cells into a senescent state, they were treated with 0.3 µM etoposide (Etop) for 2 days. The senescence *β*-Galactosidase staining kit (Beyotime Biotechnology, Shanghai, China) was used to assess the SA-*β*-Gal activity. After SA-*β*-Gal staining was performed at 37 °C for 16 h, the stained cells were observed using a bright-field microscope (Olympus BX-63, Tokyo, Japan). Three biological replicate groups with approximately 150 cells were measured in the field. The number of aging cells with SA-*β*-Gal blue stain was quantified using ImageJ software (version 1.42q, National Institutes of Health, Rockville, MD, USA). To ensure unbiased quantification, the experiment was performed under blinded conditions. The analysis was performed by an observer blinded to treatment conditions. Quantify blue-stained positive cells using ImageJ software. The threshold parameters are manually set based on representative positive control images and then applied to all images in the same batch to ensure consistency. Then, total cells in each field were manually counted, and the number of blue-stained positive cells was recorded. Finally, the percentage of SA-*β*-Gal positive cells was calculated as (number of blue-stained positive cells/total cells) × 100%.

### 2.5. Antioxidant Stress Experiment

BY4741 yeasts with 0.01 value of OD_600_ were cultured in 20 mL YPD medium, which respectively contained DISS at doses of 0, 1, 3, 10 μM or 10 μM RES, and were cultured in a 180 rpm shaker at 28 °C. Subsequently, the YPD agar plates with 5.5 mM H_2_O_2_ were spotted with 5 µL yeast culture, which was diluted 10 times by PBS. Growth was observed daily and photographed after 48 h. To digitize the antioxidant stress results of DISS, approximately 200 yeast cells were spread onto a YPD agar plate with or without 4.5 mM H_2_O_2_, and incubated for 48 h. The survival rate of the cells was calculated as the ratio of the number of colonies growing on the H_2_O_2_-containing medium to that growing on the H_2_O_2_-free medium.

### 2.6. Determination of ROS and MDA Levels

The culture of BY4741 yeast was the same as described in the antioxidant stress experiment section. After that, the yeast cultures were centrifuged to remove the medium and washed with PBS. Subsequently, yeast cells were loaded with fluorescent probe 2′, 7′-dichlorodihydrofluorescein diacetate (DCFH-DA) at a final concentration of 10 µM, and incubated with shaking in the dark for 60 min. To clear excess DCFH-DA in the medium, the yeast cells were washed with PBS three times and resuspended in 220 µL PBS. The 200 µL suspension of yeast cells in each group was added to a 96-wells black plate, respectively. Under the condition of an excitation wavelength of 488 nm and an emission wavelength of 525 nm, the fluorescence intensity of DCF of 1 × 10^7^ cells was detected by the BioTek microplate reader (BioTek, Winooski, VT, USA). The cell number per well was determined using a hemocytometer, and the raw fluorescence values were normalized accordingly. ROS level = (Sample fluorescence − Blank fluorescence)/Number of cells per well (×10^7^).

In the process of MDA detection, the incubation of yeast was the same as that of the ROS assay. The yeasts were washed and ground with grinder beads at 70 Hz by an automated sample rapid grinder (Shanghai Jingxin Inc., Shanghai, China). Subsequently, the sample was centrifuged to obtain the supernatants at 12,000 rpm for 10 min at 4 °C. The BCA kit (CoWin Biotech, Beijing, China) was used to determine the concentration of the supernatants. The supernatants were diluted to 1.25 µg/µL and used as protein samples. The level of MDA of yeast was determined by the MDA assay kit (Nanjing Jiancheng Bioengineering Institute, Nanjing, China) according to the manufacturer’s instructions. MDA content (nmol/mg protein) = {(Absorbance of the sample − Absorbance of the blank)/Absorbance of the standard tube} × Standard concentration/Total protein in the sample.

### 2.7. Determination of SOD, CAT, and GPx Antioxidant Enzyme Activities

In the test for antioxidant enzyme activities, the procedures for yeast culture, protein extraction and measurement were similar to those used in the MDA assay. Each sample’s protein concentration was diluted to 1.25 µg/µL for the determination of antioxidant enzyme activity. The SOD assay kit (Nanjing Jiancheng Bioengineering Institute, Nanjing, China), the CAT and GPx assay kits (Beyotime Biotech, Shanghai, China) were used to measure the activity of T-SOD, CuZn-SOD, CAT and GPx as described in Supplementary Information Section S1.2, respectively.

### 2.8. Real-Time Fluorescent Quantitative PCR (qRT-PCR)

S288C yeasts with 0.01 value of OD_600_ were cultured in 20 mL YPD media, which respectively contained DISS at doses of 0, 1, 3, or 10 µM or 10 µM RES for 24 or 48 h. Then, the yeast cells were collected by centrifugation. The grinding beads and 1 mL of TRIzon reagent (CoWin Biotech, Beijing, China) were added to the yeast cells, and the mixture was ground at 68 Hz for 3 min, and then placed on ice for 10 min. Subsequently, approximately 200 μL of chloroform was added to each sample, and then the mixtures were vortexed, let stand for 5 min, and centrifuged for 15 min. The aqueous phase was transferred to new tubes, and the total RNA was precipitated with isopropanol. To get more RNA, all of the tubes containing RNA sediment were placed on ice for 10 min. Afterwards, the pellets of RNA in tubes were washed with 75% EtOH twice. After the RNA pellet was dried, it was dissolved in 30 µL of RNase-free water. Following the determination of total RNA concentration with a NanoDrop one Ultra-Micro Spectrophotometer (Thermo Scientific, Wilmington, DE, USA), cDNA was synthesized from 5 µg of RNA using the HiFi-MMLV cDNA kit (CoWin Biotech, Beijing, China). Transcripts were quantified by PCR analysis with CFX 96 Touch (BioRad, Hercules, CA, USA) and SYBR premixed EX Taq (Takara, Otsu, Japan). The primers used in this study were synthesized by Sangon Biotech Co., Ltd., Shanghai, China, and are displayed in Supplementary Table S3. The thermal recycling parameters were as follows: 95 °C for 2 min, followed by 40 cycles; 95 °C for 15 s; 55 °C for 15 s; and 68 °C for 20 s. All results were normalized to TUB1 and analyzed using the 2^−∆∆Ct^ formula.

### 2.9. Fluorescence Imaging of Autophagy

Briefly, the YOM38 yeasts containing the pRS316-GFP-ATG8 plasmid were inoculated and cultured in YPD medium for 24 h in the dark. After that, the yeast cells with 0.01 value of OD_600_ were added to a 50 mL flask with 20 mL of SD medium and treated with DISS at doses of 0, 1, 3, or 10 µM, or 10 µM RES for 24 h, respectively. Subsequently, the stain of Hoechst 33342 was added to the culture medium at a final concentration of 1 µg/mL, and the cells were stained for 8 min in the dark. After that, the cells were washed with PBS to remove the redundant stain. Finally, a confocal fluorescence microscope (Olympus BX-51, Tokyo, Japan) was used to observe the free GFP with 488 nm wavelength and a 60× oil immersion objective, and to take the micrographs. Each group was selected to take micrographs of nine visual fields, the 300–350 cells of each group were quantified for analysis, and those exhibiting free GFP were scored as positive.

### 2.10. Evaluation of Telomerase Content in Yeast

At first, the S288C yeast cells with 0.01 value of OD_600_ were added to 20 mL of YPD medium containing DISS at doses of 0, 1, 3, 10 μM or 10 µM RES in 50 mL flasks and cultured for 48 h. Subsequently, the yeast cells were collected and ground with grinding beads at 70 Hz for 3 min. The sample was centrifuged at 12,000 rpm for 10 min at 4 °C, and the supernatants were used as protein samples. Meanwhile, the BCA kit was used to measure protein concentrations and each sample was diluted to 10 µg/µL. Telomerase ELISA kit (Shanghai Tongwei Company, Shanghai, China, Cat#TW11187) was used to measure the telomerase content of the yeasts as described in a previous study [21]. Briefly, the microtiter wells containing the telomerase antibody in the kit were sequentially filled with 50 µL blank (sample diluent), 50 µL treated-group protein (20 µL protein sample plus 30 µL sample diluent), or 50 µL standards with different concentrations (12.5, 25, 50, 100, or 200 U/L) and incubated for 30 min at room temperature. After that, the liquid in each well was aspirated and washed with the scrubbing solution three times. Subsequently, the 50 µL horseradish peroxidase-labeled antibody was added to each well, respectively. All of the wells were incubated in a warm bath until a color change from blue to yellow, and the intensity of the color was measured at 450 nm wavelength with a BioTek microplate reader (BioTek, Winooski, VT, USA). The standard curve, created by plotting the average optical density obtained for six standard concentrations on the vertical (Y) axis, was used to determine the telomerase amount in the samples.

### 2.11. Measurement of Relative Telomere Length

In this experiment, the mammalian genomic DNA extraction kit (Beyotime Biotechnology Co., Ltd., Shanghai, China) was used to obtain the DNA for 3T3 cells, which were seeded in 10 mL CM sodium and treated with 0, 1, 3, 10 μM DISS or 10 μM AST for 48 h at 37 °C. Subsequently, the DNA concentration was determined by a NanoDrop one ultra-micro spectrophotometer, and the agarose gel electrophoresis was used to detect genomic DNA quality, respectively. Then, the relative telomere length of 3T3 cells in this study was measured by the Relative Human Telomere Length Quantification qPCR (ScienCell Research Laboratories, San Diego, CA, USA). This approach is methodologically valid as the kit’s single-copy reference (SCR) primer set specifically recognizes a 100 bp region on mouse chromosome 10 for accurate normalization, while the telomere primers efficiently amplify the highly conserved (TTAGGG) repeats in mouse telomeric DNA. The 20 μL detection system contained target DNA for 2 ng, two pairs of primers, 2 × Golden Start TaqGreen qPCR Master Mix (ScienCell Research Laboratories, San Diego, CA, USA) and RNase-free water. The PCR procedure is recorded in Supplementary Table S4. The PCR experiment was conducted on CFX 96 Touch (BioRad, Hercules, CA, USA), and the Cq values were obtained for each group. The relative telomere length was calculated according to the provided formula: ∆Cq (SCR) = Cq (Sample, SCR) − Cq (Control, SCR); ∆Cq (TEL) = Cq (Sample, TEL) − Cq (Control, TEL); ∆∆Cq = ∆Cq (TEL) − ∆Cq (SCR); relative telomere length (Sample to Control) = 2^−∆∆Cq^.

### 2.12. Drug Affinity Responsive Target Stability (DARTS) Assay

The DARTS experiment was performed as in the previous literature [20]. A total of 2 × 10^6^ 3T3 cells were washed three times with PBS and collected in Eppendorf tubes. Then, the 200 μL RIPA buffer (Beijing CoWin Biotech Co., Ltd., Beijing, China) containing 1% protease inhibitor and 2% phosphatase inhibitor was added to each tube and the cells were lysed for 30 min. The supernatants as protein samples were obtained by centrifugation at 12,000 rpm for 20 min at 4 °C. The concentration of protein samples was determined by BCA kit (CoWin Biotech, Beijing, China) and diluted to 2 μg/μL. Subsequently, the 50 μL of each sample was incubated with DISS at doses of 0, 1, 3, 10, 30 μM for 1 h at room temperature, respectively. Except for the control group, the protein samples from other groups were digested with pronase E (Med Chem Express, Shanghai, China, Cat#9036-06-0, Active unit ≥ 7000 U/g) dissolved in TNC buffer (50 mM Tris-HCl, pH 8.0, 50 mM NaCl, 10 mM CaCl_2_) for 15 min at room temperature. The enzyme was added to the protein sample in the ratio of 1:1000 enzyme to protein. Equal amounts of 5 × SDS-PAGE (sodium dodecyl sulfate polyacrylamide gel electrophoresis) loading buffer were added to each sample and heated at 100 °C for 8 min to quench the pronase E activity and let the protein denature. Finally, Western blot analysis was performed with SIRT1 and *β*-actin antibodies.

### 2.13. Cellular Thermal Shift Assay (CETSA)

The CETSA procedure was carried out as previously reported [20]. The protein preparation was the same as the above section. First, the protein samples were incubated with 0 and 10 μM DISS for 2 h at room temperature, and each group was divided into 5 aliquots. Subsequently, samples were heated for 3 min at temperatures ranging from 37 to 70 °C. In the isothermal dose-reaction experiment, the supernatant of 3T3 cell lysis was incubated with 0, 1, 3, 10 or 30 μM DISS for 2 h at room temperature and heated at 60 °C for 3 min. After that, the samples were centrifuged, and the supernatants were collected. Each sample was mixed with 5× SDS-PAGE loading buffer at a ratio of 4:1, and heated for 8 min at 100 °C for Western blot analysis with SIRT1 and *β*-actin antibodies.

### 2.14. Western Blot Analysis

Western blot analysis was conducted according to the methodology described in a previous study [21]. In the time-course experiment, YOM38 yeast was treated with 1 µM DISS for 0, 8, 15 and 22 h. The observation point was chosen as 22 h. In the dose-course experiment, YOM38 yeast was treated with 0, 1, 3 or 10 µM DISS for 22 h. Additionally, 3T3 cells were treated with 10 µM AST or 0, 1, 3 or 10 µM DISS for 48 h. Protein was extracted from harvested cells from all treatment groups, and the protein concentration was measured using a BCA kit. After that, about 20 μg protein sample of each group was loaded onto a sodium dodecyl sulfate polyacrylamide gel and separated under conditions of constant voltage. Subsequently, the sample was transferred to a polyvinylidene fluoride (PVDF) membrane under conditions of constant current. The membrane was incubated with primary antibody (anti-SIRT1, anti-GFP, anti-p53, anti-p21, anti-TRF2 and anti-RAP1, anti-*β*-actin) at 4 °C for overnight, respectively. After that, the membrane was incubated with the secondary antibody, goat anti-rabbit IgG or goat anti-mouse IgG with HRP for 45 min at room temperature. The membrane was imaged with a multifunctional molecular imaging system (Vilber Bio Imaging, Paris, France) and an ECL Western blot kit (CoWin Biotech, Beijing, China), and blot density was quantified using ImageJ Lab software (version 6.1, Bio-Rad, Hercules, CA, USA).

### 2.15. Statistical Analysis

Experimental data was analyzed by GraphPad Prism 9.5.0 software (GraphPad Software, San Diego, CA, USA). For CLS analysis, we used log rank test to analyze the survival curves between the control group and the treatment group. For other data, we used one-way ANOVA and Tukey’s post hoc test. Prior to the analysis of variance, we validated the hypothesis that the data conforms to normality and homogeneity of variance. The data of the experiments were shown as the mean ± SEM. The *p* < 0.05 represents a significant difference compared to the negative control group.

## 3. Results

### 3.1. DISS Prolongs the Lifespan of Yeast and Relieves Etop-Induced Senescence of Mammalian Cells

*Saccharomyces cerevisiae* is an important model organism for aging research, widely used in high-throughput screening and the mechanism of action of anti-aging drugs. In this study, the anti-aging effects of DISS were evaluated by replicative and chronological lifespan assay in yeast. Resveratrol and rapamycin have been established as positive controls due to their clear mechanisms in Sirtuin activation and mTOR inhibition, as well as their reproducible longevity effects [3,12]. As we expected, DISS and RES extended the replicative lifespan of K6001 yeast, and DISS and Rapa extended the chronological lifespan of BY4741 yeast, respectively (Figure 1B,C and Supplementary Figure S2). In the replicative lifespan assay, the average lifespan of the control group and RES group was 5.40 ± 0.22 and 9.18 ± 0.50 (*p* < 0.001). The average lifespan of 1, 3, and 10 μM DISS group was 7.45 ± 0.55 (*p* < 0.05), 8.35 ± 0.69 (*p* < 0.001), 8.78 ± 0.51 (*p* < 0.001). Meanwhile, the mean chronological lifespan of DISS groups was extended from 17 days (Control) to 21 days (3 μM DISS) and 23 days (10 μM DISS). Interestingly, the maximum lifespan of DISS groups was increased from 31 days to 39 days, respectively. Furthermore, the anti-aging effects of DISS on mammal cells were confirmed in 3T3 cells. Etoposide is an inhibitor of topoisomerase II, which prevents the double-stranded DNA from reconnecting, leading to sustained genomic damage and cell death [22]. Therefore, it was used to construct the aging model at the cell level. After drug treatment, the percentage of aging cells with SA-*β*-Gal blue stain in the Etop-treated group was significantly increased from 10.02% ± 0.92% to 60.86% ± 1.31% compared with the control group (Figure 1D,E, *p* < 0.001). Meanwhile, the percentages of SA-*β*-Gal senescent cells in the Rapa and DISS groups were decreased to 32.75% ± 1.58% (50 nM Rapa), 56.25% ± 1.40% (1 μM DISS), 54.77% ± 1.28% (3 μM DISS), 48.07% ± 0.25% (10 μM DISS) compared with the Etop-treated group (Figure 1D,E, *p* < 0.001, *p* > 0.05, *p* < 0.01, *p* < 0.001), respectively. These results suggested that DISS from *Polygalae Radix* had anti-aging effects on yeast and mammal cells.

### 3.2. DISS Potentially Targets SIRT1 and Regulates SIRT1/p53/p21 Pathway

**Figure 2:**
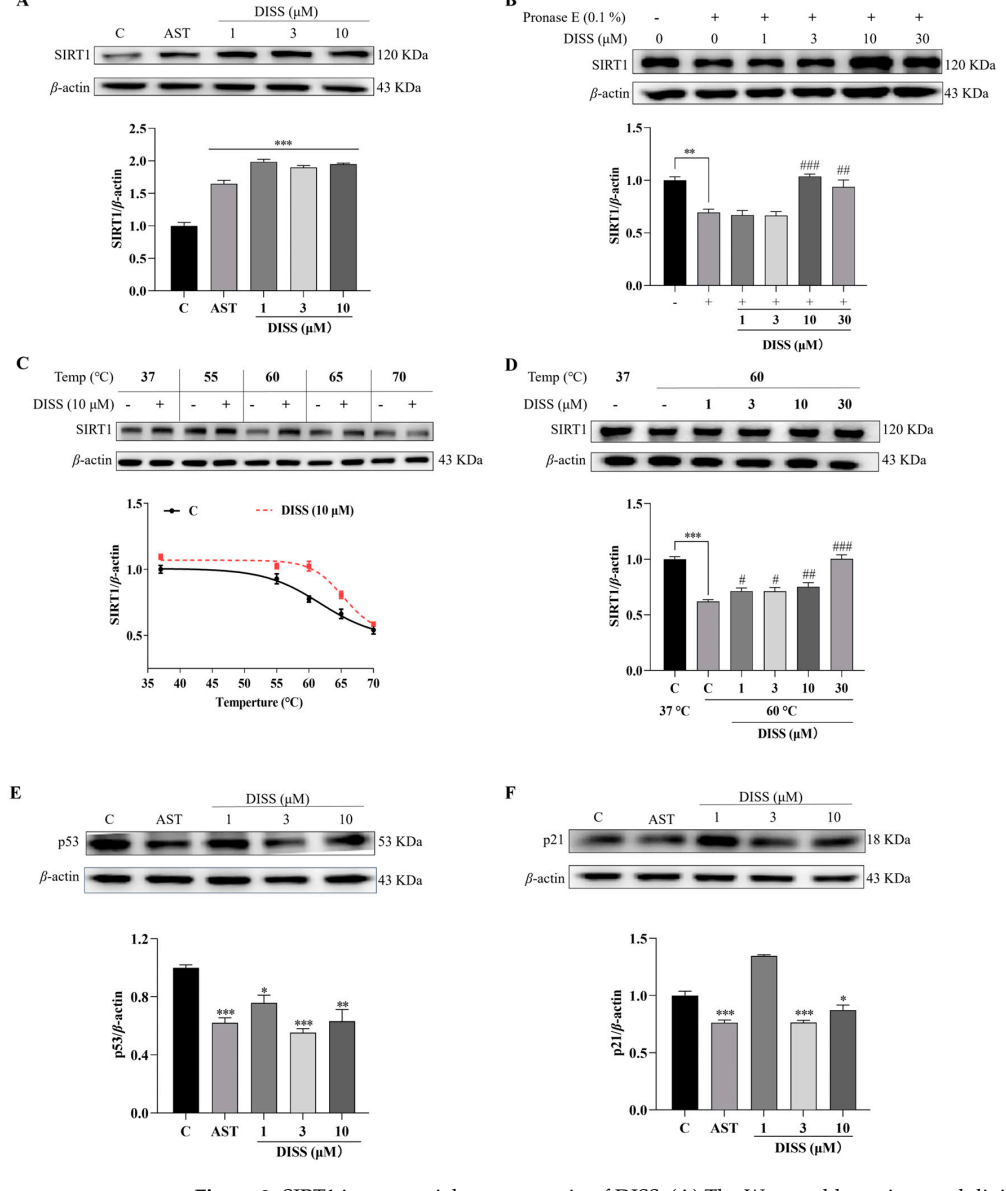
SIRT1 is a potential target protein of DISS. (**A**) The Western blot stripes and digital results of SIRT1 protein in 3T3 cells after incubation with 10 µM AST or 0, 1, 3 and 10 µM DISS. (**B**) The SIRT1 expression and digital results in the DARTS experiment. (**C**,**D**) The SIRT1 expression and digital results in the CESTA experiment. (**E**) The p53 expression and digital results after giving 10 µM AST or DISS at doses of 1, 3, 10 μM. (**F**) The p21 expression and digital results after giving 10 µM AST or DISS at doses of 1, 3, 10 μM. Western blot analysis was performed with three biological replicates. (*n* = 3). *, ** and *** represent significant differences at *p* < 0.05, *p* < 0.01, and *p* < 0.001 compared with negative control group, respectively. ^#^, ^##^ and ^###^ represent significant differences at *p* < 0.05, *p* < 0.01 and *p* < 0.001 compared with the negative control group that was treated with pronase E or temperature, respectively.

SIRT1 affects telomere length and stability by regulating telomerase activity, DNA repair and cell metabolism, thus delaying cell aging. Specifically, it can stabilize telomere protective proteins, such as tripeptidyl peptidase 1 (TPP1), and reduce telomere shortening rate through deacetylation [10]. Astragaloside IV, which can maintain telomere function at the cellular level, was selected as a positive control [21]. Thus, the changes in specific proteins in the SIRT1 signaling pathway were first investigated with Western blot analysis. The significant increase in SIRT1 protein level was observed in all of the DISS groups (Figure 2A and Supplementary Figure S3, *p* < 0.001, *p* < 0.001 and *p* < 0.001). The specific interaction between small molecule drugs and intracellular targets is the basis for their pharmacological activity [23]. Thus, the discovery and identification of the target protein for small molecular compounds was an important link for new drug development. DARTS and CETSA are two non-targeted methods used to identify the target proteins of small molecular drugs. The characteristic of DARTS is that if the affinity between small molecule drugs and proteins is stronger, then the stability of proteins is better. CETSA technology is the other approach that confirms the target proteins according to the thermal stability of small-molecule compounds and their binding proteins [23]. Therefore, the two methods were utilized to discover the target protein of DISS. In the DARTS experiment, the SIRT1 protein level in the pronase E-treated group was significantly decreased compared with the control group. Meanwhile, DISS significantly increased the stability of SIRT1 protein in the group treated at an enzyme-to-protein mass ratio of 1:1000 (Supplementary Figure S4). In the dose-course experiment, the stability of SIRT1 protein against pronase E was increased after adding DISS at doses of 10 and 30 μM (Figure 2B and Supplementary Figure S3, *p* < 0.001 and *p* < 0.01). Furthermore, CETSA was also performed to detect the changes in SIRT1 protein under heating conditions within a certain temperature range. As expected, the decrease and increase in SIRT1 in control groups and in DISS groups were observed (Figure 2C,D and Supplementary Figure S3), respectively. These evidences indicated that SIRT1 may be the potential target protein of DISS to produce anti-aging effects. Since SIRT1 modifies p53 by deacetylation to inhibit the expression of p21 and cell senescence, the changes in p53 and p21 proteins after giving DISS were also detected. The expressions of them in the AST group and DISS-treated group at doses of 3 and 10 μM were lower than those of the control group (Figure 2E,F and Supplementary Figure S3), respectively. These evidences indicated that SIRT1 was the potential target protein of DISS, and SIRT1/p53/p21 signaling pathway was involved in the anti-aging effects of DISS.

### 3.3. DISS Increases the Telomerase and Length of Telomeres

**Figure 3:**
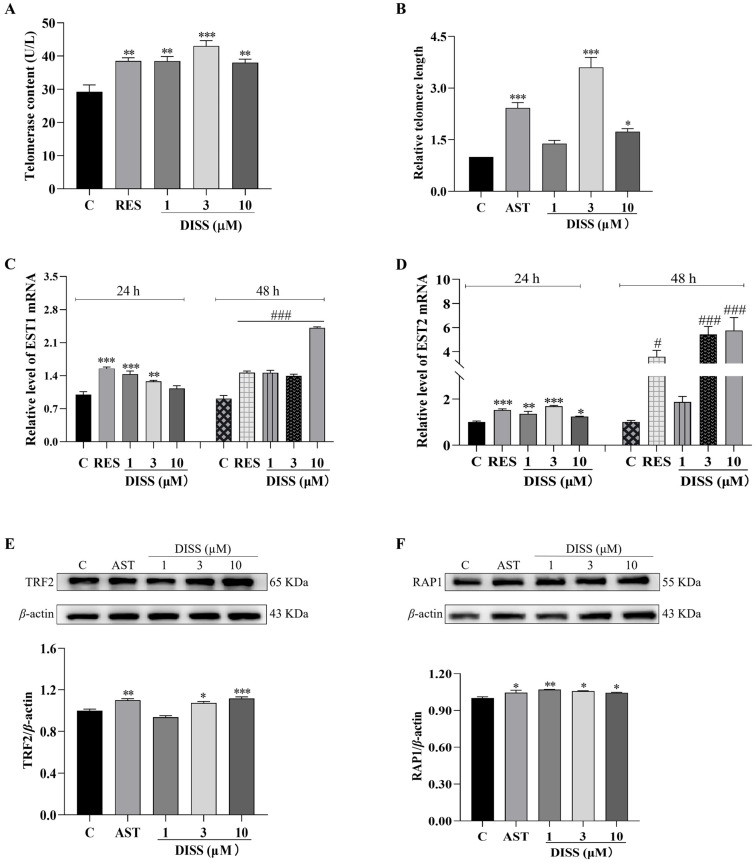
Effect of DISS on telomere. (**A**) The changes in telomerase content after treatment with 0, 1, 3, 10 μM DISS or 10 μM RES. (**B**) Relative telomere length of 3T3 cells after treatment with 0, 1, 3, 10 μM DISS or 10 μM AST. (**C**,**D**) Effects of 1, 3, 10 μM DISS or 10 μM RES on *EST1*, *EST2* genes at 24 and 48 h. (**E**) Effects of 0, 1, 3, 10 μM DISS or 10 μM AST on TRF2 protein expressions and digital results. (**F**) Effects of 0, 1, 3, 10 μM DISS or 10 μM AST on RAP1 protein expressions and the digital results. qPCR was performed for 40 cycles. For qPCR, each RNA sample was subjected to three technical replicates (*n* = 3). Western blot analysis was performed with three biological replicates. (*n* = 3). *, ** and *** represent significant differences at *p* < 0.05, *p* < 0.01, and *p* < 0.001 compared with the negative control group, respectively. ^#^ and ^###^ represent significant differences at *p* < 0.05, *p* < 0.001 compared with the negative control group for 48 h, respectively.

Telomeres can keep the stability of chromosome ends and prevent DNA degradation and abnormal recombination. In addition, the telomeres shorten with each cell division and under oxidative stress, and telomere damage is an important hallmark of normal aging [4]. Therefore, we detected the effect of DISS on telomerase activity in yeast and telomere length in mammalian cells. As shown in Figure 3A, a significant increase in telomerase in the RES and DISS groups was observed compared with the negative control group. The amount of telomerase in the RES group was increased from 29.25 ± 2.04 U/L to 38.50 ± 0.96 U/L (*p* < 0.01), and those of the DISS group at doses of 1, 3 and 10 μM were augmented to 38.49 ± 1.37 U/L (*p* < 0.01), 43.00 ± 1.63 U/L (*p* < 0.001), and 38.04 ± 1.01 U/L (*p* < 0.01), respectively. Moreover, the effect of DISS on telomere length was further explored in 3T3 cells. After treating with DISS at all doses, the telomere length of 3T3 cells in DISS groups at doses of 3 and 10 μM was increased by 260.34% and 73.01% compared with the negative control group, and the effect of 3 μM DISS is optimal and comparable to that of the positive control AST (Figure 3B).

To investigate whether the effect of DISS on telomerase is related to telomerase-related genes, such as *EST1*, *EST2*, and *EST3*. The transcriptional changes in them were measured using qRT-PCR after giving DISS at different concentrations. After treatment with DISS for 24 and 48 h, the gene expressions of *EST1* and *EST2* were significantly increased compared with the control group (Figure 3C,D). However, the change in *EST3* expression in the DISS-treated group at the two time points was not observed (Supplementary Figure S5).

To understand how DISS protects telomeres, the expression of the specific protein related to telomeres, telomeric-repeat binding factor 2 (TRF2) and repressor activator protein 1 (RAP1) proteins of the shelterin protein family, was detected, respectively. As shown in Figure 3E,F, the protein expression of TRF2 was significantly increased in DISS group at doses of 3 and 10 μM (Figure 3E and Supplementary Figure S6, *p* < 0.05, *p* < 0.001). At the same time, RAP1 protein expression was increased in all of the DISS groups (Figure 3F and Supplementary Figure S6, *p* < 0.01, *p* < 0.05, *p* < 0.05), respectively. The above results supported that DISS generates anti-aging effects via the regulation of EST, TRF2 and RAP1 expression to increase telomerase and telomere length.

### 3.4. DISS Enhances the Antioxidant Capacity of Yeast

**Figure 4:**
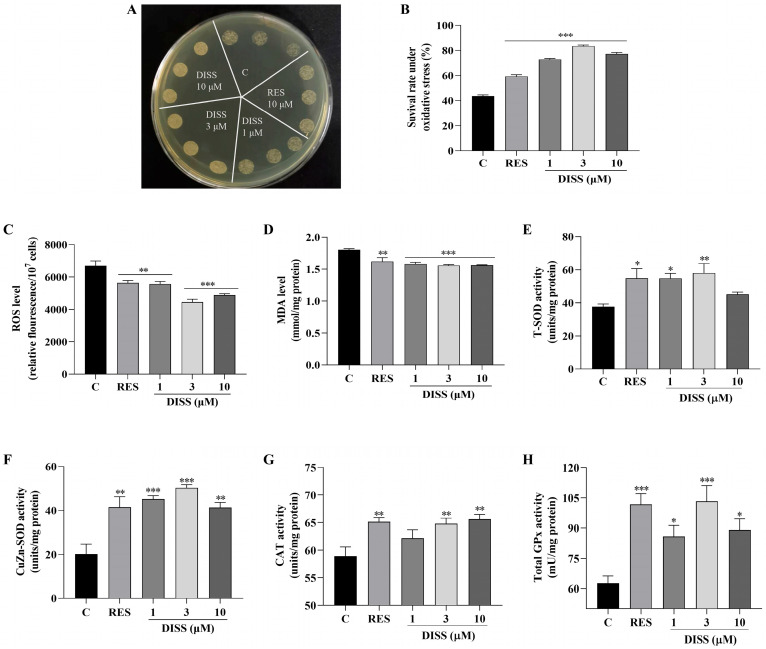
Effect of DISS on antioxidant capacity of yeast. (**A**) The growth status of BY4741 yeast under oxidative stress induced by 5.5 mM H_2_O_2_. (**B**) Quantification of yeast cell viability under 4.5 mM H_2_O_2_-induced oxidative stress. (**C**,**D**) ROS and MDA levels in yeast cells and (**E**–**H**) the effects on T-SOD, CuZn-SOD, CAT and GPx antioxidant enzyme activities in BY4741 yeast treated with 1, 3, and 10 µM DISS or 10 µM RES for 24 h. The above experiments were performed with three technical replicates. (*n* = 3). *, ** and *** represent significant differences compared with the negative control group for *p* < 0.05, *p* < 0.01, and *p* < 0.001, respectively.

As a key protein, SIRT1 has multiple functions in cells, which are crucial for health and longevity by regulating gene expression, maintaining cell homeostasis and resisting oxidative stress [7]. Thus, the effect of DISS on oxidative stress was investigated with anti-oxidative stress experiment. BY4741 yeast was used to test whether DISS can improve yeast survival under oxidative stress conditions induced by H_2_O_2_ at concentrations of 4.5 and 5.5 mM, respectively. DISS at doses of 1, 3 and 10 μM and RES at a dose of 10 μM significantly increased yeast colony numbers under oxidative stress conditions (Figure 4A). In the quantitative analysis, the survival rates of each group were 43.62 ± 1.51% in the negative control group, 59.26 ± 2.19% in the positive control group, 72.87 ± 1.24% in the 1 μM DISS group, 83.47 ± 1.34% in the 3 μM DISS group, and 77.27 ± 1.91% in the 10 μM DISS group (Figure 4B), respectively. Furthermore, the biomarkers of oxidative stress, such as ROS and MDA were also detected. The level of ROS was significantly reduced after treatment with DISS at doses of 1, 3 and 10 μM (Figure 4C). The ROS levels of treated groups were decreased from 6693.43 ± 300.18 of the control group to 5642.68 ± 146.02 of RES group, 5575.95 ± 153.31 of 1 μM DISS-treated group, 4459.50 ± 170.04 of 3 μM DISS-treated group and 4892.51 ± 77.78 of 10 μM DISS-treated group, respectively. At the same time, MDA levels in the DISS-treated group were also reduced compared with the negative group (Figure 4D). The MDA levels in the positive group decreased from 1.79 ± 0.02 to 1.62 ± 0.07, as well as those of the 1, 3 and 10 μM DISS-treated group were decreased to 1.58 ± 0.03, 1.56 ± 0.02 and 1.56 ± 0.01, respectively. To understand whether the anti-oxidative enzymes were involved in the regulation of anti-oxidative stress of DISS, we measured the changes in relative enzymes such as SOD, CAT and GPx. As shown in Figure 4E–H, the activity of T-SOD, CuZn-SOD, CAT and GPx in yeast was significantly increased after treatment with DISS and RES, respectively. These results suggested that DISS extended lifespan via modification of oxidative stress by increasing anti-oxidative enzymes and reducing ROS and MDA levels.

### 3.5. DISS Cannot Prolong the Lifespan of Yeast Lacking SOD1, SOD2, CAT, and GPx Genes

**Figure 5:**
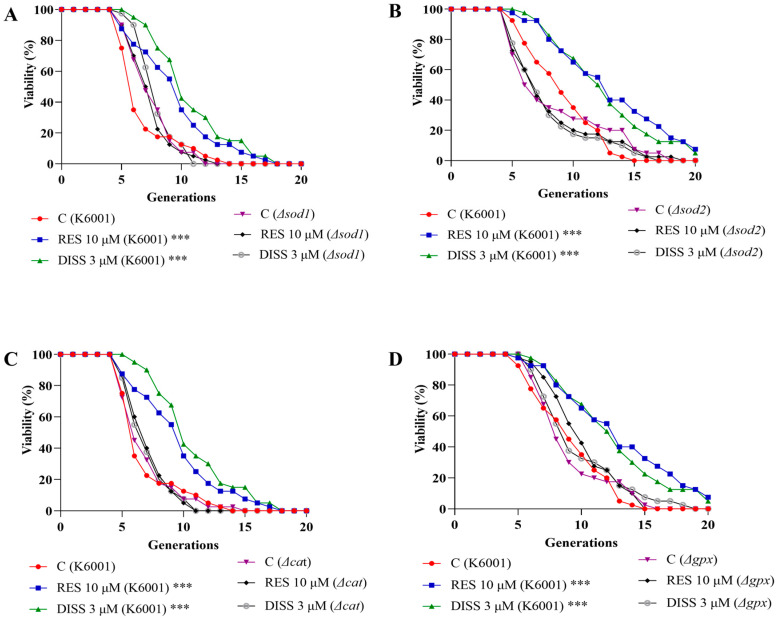
Effect of DISS on replicative lifespan of K6001 yeast mutants *Δsod1* (**A**), *Δsod2* (**B**) *Δcat* (**C**), and *Δgpx* (**D**). For the RLS, viability was measured using three biological replicates. (*n* = 3). *** represent significant differences compared with the negative control group for *p* < 0.001.

**Figure 6:**
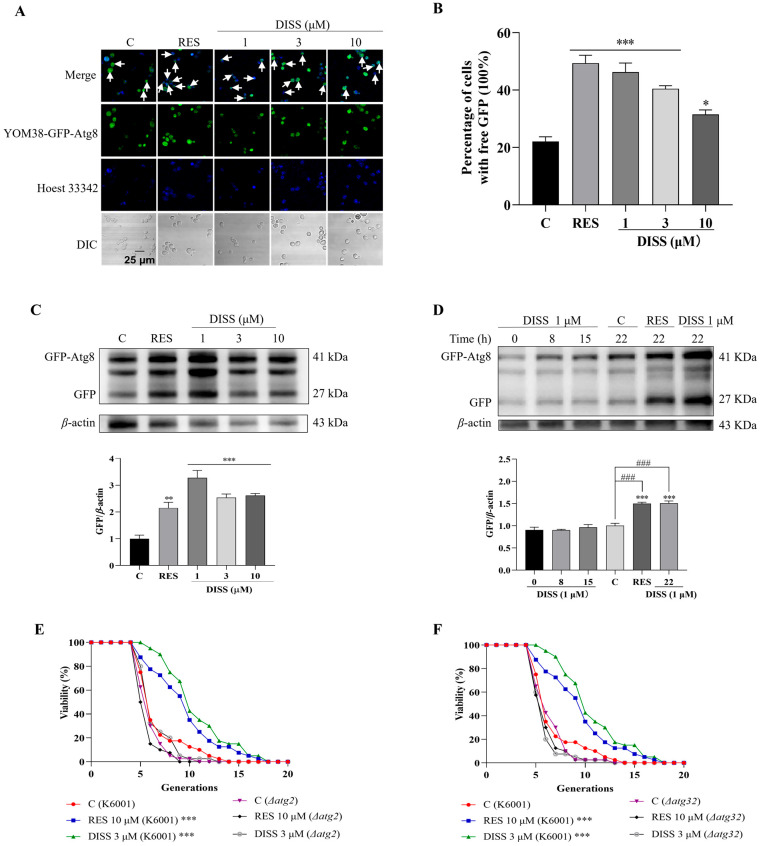
Effect of DISS on autophagy in YOM38 yeast. (**A**) Fluorescence images of autophagy after treatment with 300 µM RES or 1, 3, or 10 µM DISS for 22 h, and white arrowheads point to yeast cells undergoing autophagy. (**B**) Digital results of A. (**C**) The Western blot stripes and digitized results of free GFP in yeast cells after treatment with 300 µM RES or different doses of DISS for 22 h. (**D**) The western blot tripes and digitized results of free GFP in yeast cells treated with 300 µM RES or 1 µM DISS for 0, 8,15 or 22 h. (**E**,**F**) Replicative lifespan of K6001 yeast and mutants *Δatg2* (**E**) and *Δatg32* (**F**) treated with 10 µM RES or 3 µM DISS. The average lifespan in K6001 was 5.98 ± 0.38 in the negative control group, 8.73 ± 0.54 in the positive treatment group (*p* < 0.001), and 9.93 ± 0.50 in the group treated with 3 µM DISS (*p* < 0.001). (**E**) The average lifespan *Δatg2* was 5.18 ± 0.21 in the negative group, 4.83 ± 0.18 in the positive group, and 5.70 ± 0.28 in the 3 µM DISS group. (**F**) The average lifespan of *Δatg32* was 5.58 ± 0.27 in the negative group, 5.20 ± 0.26 in the positive group, and 5.08 ± 0.25 in the 3 µM DISS group. Western blot analysis was performed with three biological replicates. (*n* = 3). For the RLS, viability was measured using three biological replicates. (*n* = 3). *, ** and *** represent significant differences at *p* < 0.05, *p* < 0.01, and *p* < 0.001 compared with the negative control group, respectively. ^###^ represent significant differences at *p* < 0.001 compared with the negative control group.

To understand whether these anti-oxidative enzymes were directly involved in the anti-aging effects of DISS, we constructed the *Δsod1*, *Δsod2*, *Δcat* and *Δgpx* mutants of K6001 yeast and performed the replicative lifespan analysis. The replicative lifespan of K6001 yeast was significantly increased in the DISS-treated group (Figure 5A–D). The average lifespan in K6001 was 5.98 ± 0.38 in the negative control, 8.73 ± 0.54 in the positive treatment group (*p* < 0.001), and 9.93 ± 0.50 in the group treated with 3 µM DISS (*p* < 0.001), as shown in Figure 5A,C. The average lifespan in K6001 was 8.25 ± 0.45 in the negative control group, 12.1 ± 0.73 in the positive treatment group (*p* < 0.001), and 11.7 ± 0.65 in the group treated with 3 µM DISS (*p* < 0.001), as shown in Figure 5B,D. However, similar changes in the replicative lifespan of these mutants of yeast were not observed after giving a dose of 3 μM DISS (Figure 5A–D). The average lifespan of *Δsod1* was 6.70 ± 0.30 in the negative group, 6.60 ± 0.29 in the positive group, and 7.13 ± 0.24 in the 3 µM DISS group (Figure 5A). The average lifespan of *Δsod2* was 7.63 ± 0.67 in the negative group, 7.28 ± 0.59 in the positive group, and 7.13 ± 0.53 in the 3 µM DISS group (Figure 5B). The average lifespan of *Δcat* was 6.05 ± 0.36 in the control group, 6.28 ± 0.27 in the positive group, and 6.18 ± 0.28 in the 3 µM DISS group (Figure 6C). The average lifespan of *Δgpx* was 8.18 ± 0.47 in the control group, 9.25 ± 0.42 in the positive group, and 8.9 ± 0.54 in the 3 µM DISS group (Figure 5D). Overall, the SOD1, SOD2, CAT and GPx took important roles in the anti-aging effects of DISS.

### 3.6. DISS Enhances Autophagy of Yeast

SIRT1 has a close regulatory relationship with autophagy, mainly by promoting autophagy flow and regulating autophagy-related protein expression to affect autophagy substrate degradation, thereby exerting its effect [7]. To understand how DISS regulates autophagy, we used the YOM38-GFP-ATG8 yeast strain, which expressed the GFP-Atg8 fusion protein, to measure the effect of DISS on autophagy. In autophagy, free GFP is released through the vacuolar protease-mediated hydrolysis of the GFP-Atg8 fusion protein [21]. After treatment of RES and DISS, the significant increase in free GFP in the yeast can be clearly observed, respectively (Figure 6A,B, *p* < 0.001, *p* < 0.001, *p* < 0.001, *p* < 0.05). Furthermore, we first performed the investigation of the dose and time relationship of DISS with Western blot analysis. The expression of free GFP protein was significantly increased in all of the groups after incubation with DISS for 22 h, and the dose of 1 µM DISS is the optimum concentration (Figure 6C,D and Supplementary Figure S7). To explore whether the longevity of yeast cells is due to autophagy, we used two kinds of K6001 yeast mutants of *Δatg2* and *Δatg32* associated with autophagy to do a replicative lifespan assay (Figure 6E,F). As expected, the extended lifespan effect of DISS for K6001 yeast disappeared in these mutants. These results suggested that DISS exerted anti-aging effects via modifying autophagy.

## 4. Discussion

Traditional Chinese medicine is a form of medicine developed by the Chinese nation through a long history of struggling with diseases. It has not only made indelible contributions to the survival and reproduction of the people of all ethnic groups in history, but also plays an irreplaceable role in improving the health level of the people and promoting the coordinated development of economy and society under today’s historical conditions. In particular, the treatment of traditional Chinese medicine emphasizes syndrome differentiation, and people with different constitutions have different treatment methods for the same disease. This holistic conditioning model makes up for the limitations of single-target drugs and provides a new path for long-term health management. The application of new modern science and technology in traditional Chinese medicine has achieved some success. The discovery of the mechanism by which berberine regulates intestinal flora reveals the mechanism of microecological intervention of traditional Chinese medicine [2]. Nano targeting technology of traditional Chinese medicine improves the bioavailability [24]. The composition analysis of Artemisia annua and the discovery of artemisinin have created a new approach in natural medicinal chemistry [25]. Therefore, it is an important resource for new drug development.

*Polygalae Radix* is one of the traditional Chinese medicines with many chemical components. Some evidence has reported that it not only has anti-oxidative, anti-cancer, and anti-depressant effects [17,18], but also has the functions of neuronal protection and improvement of memory dysfunction [17]. In this study, we focused on the material basis and the mechanism of the anti-aging effects of *Polygala Radix* to conduct research work. At first, we utilized the separation technology guided by the bioactivity assay system, the replicative lifespan assay of K6001 yeast, to search for anti-aging compounds from this medicine and determine the chemical structures of these compounds. The DISS, as the most important compound with anti-aging effect in Figure 1 was found, and the anti-aging effects of DISS at a dose of 3 μM were equal to that of RES at a dose of 10 μM.

SIRT1, also known as silencing information regulatory factor 2-related enzyme 1, is the most representative member of the sirtuin protein family [4]. SIRT1 has acetyltransferase activity, which can remove acetyl groups from lysine residues of target proteins. This modification can significantly alter the activity, stability, cellular localization, or interactions with other molecules of target proteins, thereby regulating cellular life activities [7]. The identification of target proteins is a crucial step to reveal the mechanism of action. Since SIRT1 plays important roles in anti-aging, we hypothesized that SIRT1 was a possible target protein of DISS. To indicate this conjecture, we first investigated whether SIRT1 was affected by DISS and determined whether SIRT1 was the target protein of DISS, using the discovery methods for non-modified target molecules, such as DARTS and CETSA. Furthermore, the proteins p53 and p21, which are located downstream of the SIRT1 signaling pathway, were examined, respectively. The increase in SIRT1 expression and its thermal stability, the reduction in SIRT1 degradation by pronase E, and the significant reduction in p53 and p21 levels in Figure 2 confirmed that SIRT1 was one of the potential target proteins of DISS, and the SIRT1/p53/p21 signaling pathway was involved in the anti-aging effects of DISS.

Telomere shortening is one of the key biological markers of cell senescence. Telomere is composed of repeated DNA sequences and related proteins [4]. Its main function is to protect chromosomes from damage in the process of replication. At each cell division, DNA replicase cannot completely replicate the ends of chromosomes, resulting in telomere shortening [4]. Some cells contain telomerase, which can directly compensate for lost end DNA sequences by increasing telomere repeat sequences [4]. Thus, we investigated the effects of DISS on telomerase of yeast and telomere length of 3T3 cells, respectively. As expected, DISS not only increased the amount of telomerase but also extended the telomere length. To understand how DISS increased telomerase and telomere length, we checked the changes in *EST1*, *EST*2, *EST3* genes related to telomerase, and TRF2, RAP1 proteins related to telomere. The increase in them after giving DISS in Figure 3 indicated that DISS increased telomerase and telomere length via enhancing expression of *EST1*, *EST2*, TRF2 and RAP1 at the gene or protein levels, respectively. The anti-aging effects of DISS are mainly achieved by protecting or stabilizing the telomere cap structure and by the activity of the enzyme itself.

SIRT1 regulates oxidative stress through reducing the secretion of pro-inflammatory factors by regulating NF-κB and other pathways, activating FOXO and other transcription factors, promoting the expression of antioxidant genes, participating in the process of DNA repair and reducing gene mutation caused by oxidative stress [7]. Thus, we also investigated whether DISS affected oxidative stress by detecting biomarkers. The changes in growth status under oxidative stress conditions, levels of ROS, MDA, and SOD1, SOD2, CAT, GPx antioxidant enzymes of yeast, and replicative lifespan of yeast mutants after giving DISS in Figure 4 and Figure 5 revealed that DISS targeted SIRT1 to inhibit oxidative stress by increasing antioxidant enzymes and reducing ROS and MDA to produce anti-aging effects.

Autophagy plays an important role in anti-aging and is regulated by SIRT1. Previous study has shown that SIRT1 promotes autophagy through post-translational modification of autophagy initiation proteins and deacetylation-dependent activation of autophagy-related transcription factors [7]. To investigate whether DISS affected autophagy by targeting SIRT1, we also detected the changes in autophagy flow in YOM38 yeast strain containing GFP-Atg8 fusion protein, and checked the relationship of autophagy and replicative lifespan with atg2 and atg32 mutants of yeast with a K6001 background, respectively. The increase in free GFP in yeast and no effect of DISS on lifespan for mutants in Figure 6 clarified that autophagy, especially mitophagy, was involved in the anti-aging effects of DISS.

RES is well known as an SIRT1 activator and an anti-aging star molecule. Recently, the evidence indicates that type 4 phosphodiesterase (PDE-4) is the target protein of RES, and it triggers a series of cellular activities by inhibiting PDE-4, including the activation of SIRT1 [26]. To date, it cannot be a new drug because of low absorption rate and bioavailability, and fast metabolism and transformation in vivo [27]. DISS is a sucrose ester derivative from *Polygalae Radix*, a traditional medicinal herb with a long history of human use. The pharmacokinetic study of *Polygalae Radix* aqueous extract (main ingredient is DISS) suggested that DISS has higher safety and better bioavailability [28]. Moreover, our finding indicates that the anti-aging effects of DISS for yeast are better than those of RES, and DISS targets the SIRT1 protein to exert anti-aging effects via regulating multi-pathway synergy. Thus, DISS, as an active lead compound, is worthy of further research and development.

In the present study, the anti-aging effects of DISS were only elucidated in yeast and mammal cell levels and only got indirect evidence for SIRT1 as the potential target protein. In addition, the bioavailability of DISS is low (only 2.36%) and it can be hydrolyzed to the secondary glycoside [28]. Therefore, we need to consider whether this compound can produce the same anti-aging effects at animal and human levels. In the future, we will improve the bioavailability of DISS via structural modification and perform systematic pharmacokinetic studies. At the same time, we used the aging animal model to check the anti-aging effects of this compound. Furthermore, we will use new technologies, such as microscale thermophoresis assay, surface plasmon resonance analysis and cryo-electron microscopy, to detect SIRT1 and DISS complex to obtain direct evidence for the target protein and take molecular docking simulations to predict the binding site of DISS on the SIRT1 protein.

## 5. Conclusions

**Figure 7:**
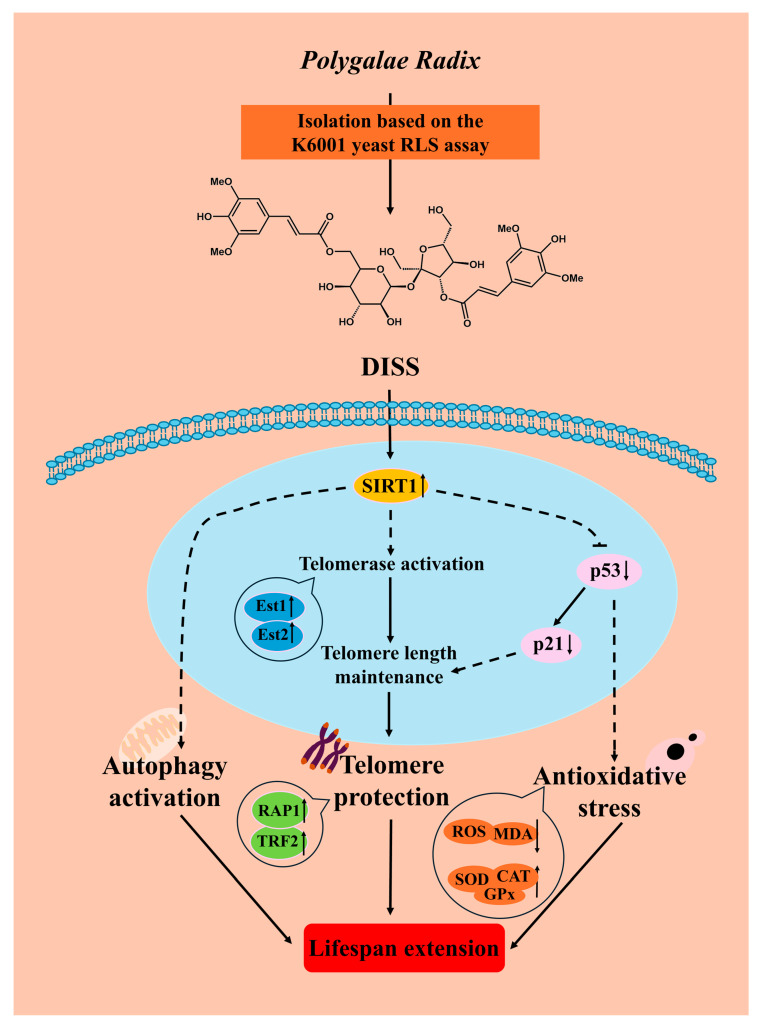
The proposed mechanism of action of DISS from *Polygala Radix* with anti-aging effects. DISS potentially targets SIRT1 and exerts anti-aging effects via regulating the p53/p21 signaling pathway, telomere, oxidative stress and autophagy. The upward black arrows indicate increase or promotion, and downward black arrows indicate decrease or inhibition.

In summary, DISS from *Polygalae Radix* targets SIRT1 as a potential target protein, regulating oxidative stress, autophagy and telomere length to exert anti-aging effects (Figure 7). This study provides important evidence for the anti-aging mechanism of DISS. At the same time, it provides a scientific basis for using *Polygalae Radix* as an anti-aging herb.

## Data Availability

The original contributions presented in this study are included in the article/Supplementary Materials. Further inquiries can be directed to the corresponding author.

## Supplementary Materials

The following supporting information can be downloaded at: https://www.mdpi.com/article/10.3390/antiox15030313/s1. Table S1. Standardized positive control conditions used in each experiment; Table S2: Yeast strains used in the present study; Table S3: PCR primer sequence; Table S4.: Relative telomere length quantification PCR procedure; Figure S1: The ^1^H NMR spectrum of DISS (500 MHz, MeOD); Figure S2. The viability of DISS in K6001 yeasts; Figure S3: Original data of Western blot analysis of the effect of DISS on SIRT1, p53, p21 and *β*-actin in Figure 2; Figure S4: The western blot stripes, digitized results and original data of western blot analysis of the effect of 10 μM DISS and different concentrations of pronase E on SIRT1 and *β*-actin; Figure S5: Effect of DISS treatment on the expression of telomerase-related genes EST3 for 24 h or 48 h; Figure S6: Original data of western blot analysis of the effect of DISS on TRF2, RAP1 and *β*-actin in Figure 3E,F. Figure S7: Original data of western blot analysis of the effect of DISS on GFP and *β*-actin in Figure 6C,D. Refs. [20,21,29,30,31,32] are cited in Supplementary Materials file.

## Abbreviations

DISS	3,6′-disinapoyl sucrose
SIRT1	Silencing information regulatory factor 2-related enzyme 1
TRF2	Telomeric repeat binding factor 2
RAP1	Repressor/activator protein 1
TPP1	Tripeptidyl peptidase 1
ROS	Reactive oxygen species
MDA	Malondialdehyde
SOD	Superoxide dismutase
CAT	Catalase
GPx	Glutathione peroxidase
RES	Resveratrol
Rapa	Rapamycin
AST	Astragaloside IV
Etop	Etoposide
RT-PCR	Real-time fluorescent quantitative PCR
RLS	Replicative lifespan
CLS	Chronological lifespan
DARTS	Drug affinity responsive target stability
CETSA	Cellular thermal shift assay
