# Supplementary material for: 3,6′-Disinapoyl Sucrose from Polygalae Radix Exerts Anti-Aging Effects via Modification of Telomeres, SIRT1/p53/p21 Pathway, Oxidative Stress and Autophagy
Source: Antioxidants (Basel). 2026 Mar 1;15(3):313. doi: 10.3390/antiox15030313 (PMC13024513; doi:10.3390/antiox15030313)
Supplement: Supplementary file 1 [file antioxidants-15-00313-s001.zip › antioxidants-4145079-supplementary.pdf]

Supplementary Information

3,6′-Disinapoyl Sucrose from *Polygalae Radix* Exerts Anti-Aging Effects via Modification of Telomeres, SIRT1/p53/p21 Pathway, Oxidative Stress and Autophagy

Jianhong Wang [1], Ting Jiang [2], Siqi Chen [1], Yajing Li [2], Qing Li [1,*], Lan Xiang [2,*] and Jianhua Qi [2]

[1] College of Chemistry and Materials Science, Sichuan Normal University, Chengdu 610068, China; 20231201025@stu.sicnu.edu.cn (J.W.); 20221201040@stu.sicnu.edu.cn (S.C.)

[2] College of Pharmaceutical Sciences, Zhejiang University, Yu Hang Tang Road 866, Hangzhou 310058, China; 22319087@zju.edu.cn (T.J.); 12019045@zju.edu.cn (Y.L.); qijianhua@zju.edu.cn (J.Q.)

* Correspondence: qingli2021@sicnu.edu.cn (Q.L.); lxiang@zju.edu.cn (L.X.)

1. Methods

1.1. Preparation of culture media

YPD medium, 2% D-(+)-glucose (Sigma-Aldrich Co., St. Louis, MO, USA), 2% hipolypeptone (Nihon Pharmaceutical Co., Ltd., Tokyo, Japan), and 1% yeast extract (Oxoid Ltd., Basingstoke, Hants, UK); YPD agar plate, add 2% ager (Sigma-Aldrich Co., St. Louis, MO, USA) to YPD medium; D-(+)-galactose liquid medium, 3% D-(+)-galactose (Sangon Biotech, Shanghai, China), 2% hipolypeptone, and 1% yeast extract; Synthetic defined (SD) medium, 2% D-(+)-glucose, 0.5% Ammonium sulfate (Xilong Chemical Co., Ltd., Guangdong, China), 0.17% Yeast Nitrogen Base (BIDI Medical Device Shanghai Co., Ltd., Shanghai, China); Synthetic complete glucose (SC)medium, add 40% L-Serine, 20% L-Threonine, 15% L-Valine, 12% L-Leucine, 10% L-Aspartic acid, 10% L-Glutamic acid, 8% L-Tryptophan, 8% L-Histidine, 8% L-Methionine, 8% Adenine, 8% Uracil, 6% L-Lysine, 6% L-Isoleucine, 6% L-Phenylalanine, 4% L-Arginine, 4% L-Tyrosine(Shanghai Aladdin Biochemical Technology Co., Ltd., Shanghai, China) to the SD medium.

1.2. Determination of SOD, GPx, and CAT antioxidant enzyme activities

The BY4741 yeasts with 0.1 value of OD_{600} was cultured in 20 mL YPD medium for 24 h, which respectively contained 0, 1, 3, or 10 μM DISS or 10 μM RES. The yeast was washed by PBS and grounded with grinder beads at 70 Hz in automated sample rapid grinder. Subsequently, the sample was centrifuged, and the concentration of the supernatant was measured by BCA Kit. The supernatant was diluted to 1.25 μg/μL and used as protein samples. The preparation of sample with SOD, GPx and CAT assay kits was carried out according to the above method. In the measurement of T-SOD enzyme activity, 2 μL protein samples and PBS were added to a 96-well plate containing Reagent I, respectively. The PBS group was used as control group. Afterwards, 6 μL reagents II, III, and IV were added to each well, and were incubated at 37 ℃ for 40 min. Finally, the samples reacted with 120 μL of color development working solution at room temperature for 10 min, and the absorbance values at 550 nm were measured. For CuZn-SOD determination, 20 μL samples and PBS were mixed with reagent VII, respectively. Then, these reacted for 1 min and centrifuged at 3500 rpm for 15 min. The supernatant was used as the protein sample. The subsequent operations as T-SOD. The activity of SOD enzyme= ([OD value of control - OD value of sample] / OD value of control) / 50% × (total volume of the reaction / sample volume) / protein concentration of sample.

In the measurement of CAT enzyme activity, the hydrogen peroxide standard curve was determined by mixing the colorimetric working solution and H_2O_2 of gradient concentrations in a 96-well plate, respectively. After incubating at 25 °C for 15 min, absorbance was measured at 520 nm. Simultaneously, hydrogen peroxide buffer, 250 mM hydrogen peroxide solution, and 10 μg protein sample were added to each well in the 96-well plate, and then reacted at 25 °C for 5 min. After that, the reaction was terminated by adding 450 μL enzyme reaction stop solution. The color development working solution mixed with 10 μL above solution mixture for 15 min and measured the absorbance at 520 nm. The activity of CAT enzyme = ([consumption of hydrogen peroxide in blank control] - [consumption of hydrogen peroxide in sample]) × [dilution ratio] / ([reaction time] × [sample volume] × [protein concentration]). For GPx enzyme activity assays, the GPx detection buffer solution and GPx detection working solution were added to each well in a 96-well plate containing 12.5 μg of protein sample, respectively. The absorbance value of the mixture was measured at every 4 min at 340 nm, and repeated six times in total. The activity of GPx enzyme = [(sample absorbance difference - blank absorbance difference)/min] / (0.00622 μM^{-1} cm^{-1} × 0.276 cm) × dilution ratio / sample protein concentration.

Supplementary Tables

Supplementary Table S1. Standardized positive control conditions used in each experiment

Experiment	Positive control	Concentration	Solvent	Time	Reason
RLS<br>ROS and MDA levels<br>CAT, SOD and GPx activities<br>Telomerase content<br>qRT-PCR<br>Antioxidant stress experiment	Resveratrol	10 μM	EtOH	Life cycle	The optimal concentration for Sirtuin activation in yeast [1-2].
CLS	Rapamycin	1 μM	EtOH	Life cycle	Yeast cells have thicker cell walls and require higher concentrations to effectively enter the cells. Yeast TORC1 has slightly lower sensitivity to rapamycin [1, 3-4].
SA-β-gal	Rapamycin	50 nM	DMSO	Life cycle	Mammalian cells are highly sensitive to rapamycin [5]. Low concentration can effectively inhibit mTORC1 [3].
SIRT1, p53, p21, TRF2<br>RAP1 expression<br>Relative telomere length	Astragaloside IV	10 μM	DMSO	Life cycle	Telomere maintenance is a long-term process that requires chronic low-dose exposure [2].
Autophagy	Resveratrol	300 μM	EtOH	Life cycle	Autophagy induction is dose-dependent, and higher concentrations used to observe excessive autophagy effects or verify autophagy pathway integrity [6].

Supplementary Table S2. Yeast strains used in the present study

Strains	Genotype	Source
K6001	MATa, ade2-1, trpl-1, canl-100, leu2-3, 112, his3-11,15, GAL, psi+, ho::HO::CDC6(at HO), cdc6:hisG, ura3::URA3 GAL-ubiR-CDC6 (at URA3)	Gifted by Professor Michael Breitenbach
Δsod1, Δsod2, Δcat, Δgpx, Δatg2, Δatg32 of K6001	Replace the SOD1 gene, SOD2 gene, CAT gene, GPx gene, ATG2 gene, and ATG32 gene in K6001 with kanamycin gene, respectively	
BY4741	MATa, his3Δ1, leu2Δ0, met15Δ0, ura3Δ0	Constructed by Professor Akira Matsuura
YOM38 containing pR316-GFP-ATG8 plasmid	Prototrophic derivative of BY4742 (MATα, his3Δ1, leu2Δ0, lys2Δ0) containing plasmid pRS316-GFP-ATG8	
S288C	MATα, SUC2, gal2, mal2, mel, flo1, flo8-1, hap1, ho, bio1, bio6	Purchased from Hangzhou Baosai Biotechnology Co., LTD

Supplementary Table S3. PCR primer sequence

Name	Forward primer	Reverse primer
Est1	5'-TTCCGTGATACCATTGGTTCTG-3′	5'-CGTCAGTGGATTACTCGTGTT-3'
Est2	5'-GGCTCAACGATCATCCTCATC-3′	5'-ATGCGACAAGTCCAATACGG-3'
Est3	5'-TTGAAGACAACTCGGAGCAT-3′	5'-ACTAAGTCAGCATCGCCAATG-3'
Tub1	5'-CCAAGGGCTATTTACGTGGA-3′	5'-GGTGTAATGGCCTCTTGCAT-3'

Supplementary Table S4. Relative telomere length quantification PCR procedure

Step	Temperature	Time	Number of cycles
Initial denaturation	95 °C	10 min	1
Denaturaiton	95 °C	20 sec	32
Annealing	52 °C	20 sec	
Extension	72 °C	45 sec	
Data acquisition		Plate read	
Optional	Melting curve analysis		1
Hold	20 °C	Indefinite	1

Supplementary Figures

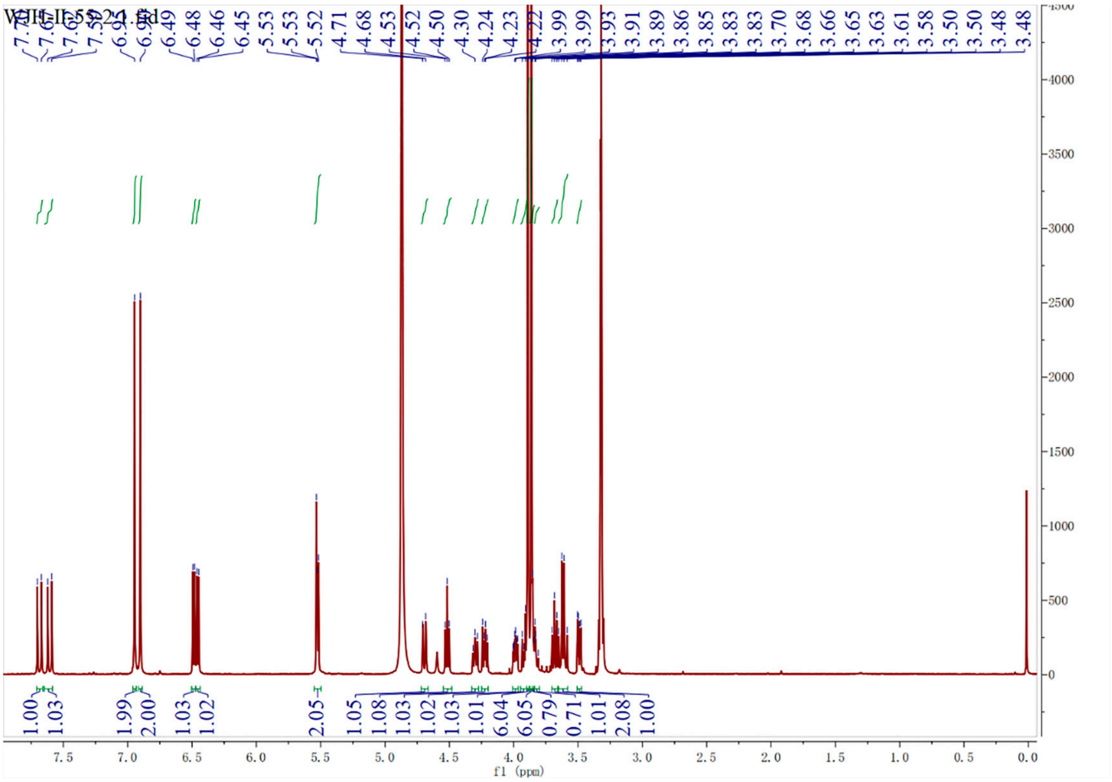

Supplementary Figure S1. The ^1H NMR spectrum of DISS (500 MHz, MeOD).

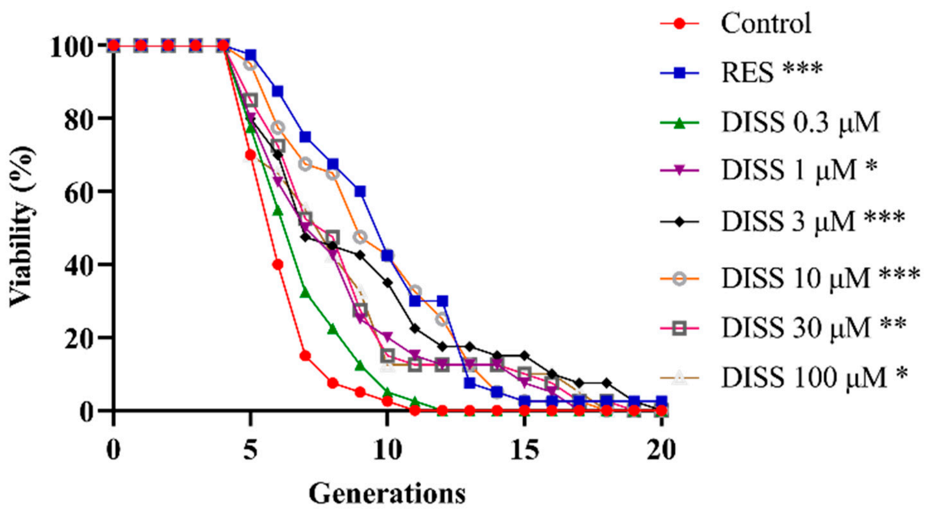

Supplementary Figure S2. The viability of DISS in K6001 yeasts.

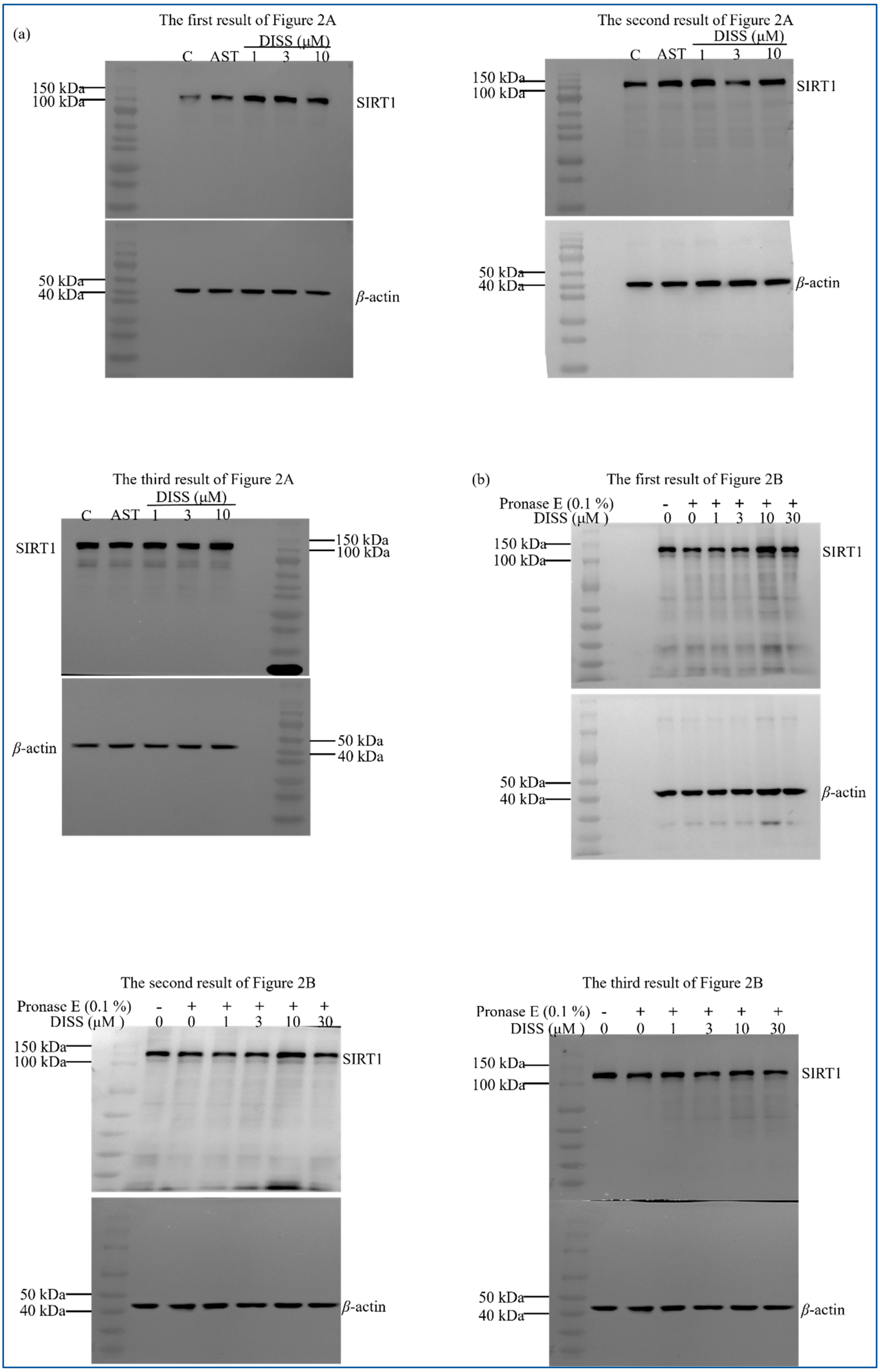
(a)
The first result of Figure 2A
DISS (μM)
C AST 1 3 10
150 kDa
100 kDa
SIRT1
50 kDa
40 kDa
β-actin
The second result of Figure 2A
DISS (μM)
C AST 1 3 10
150 kDa
100 kDa
SIRT1
50 kDa
40 kDa
β-actin
The third result of Figure 2A
DISS (μM)
C AST 1 3 10
SIRT1
150 kDa
100 kDa
β-actin
50 kDa
40 kDa
(b)
The first result of Figure 2B
Pronase E (0.1 %) - + + + + +
DISS (μM) 0 0 1 3 10 30
150 kDa
100 kDa
SIRT1
50 kDa
40 kDa
β-actin
The second result of Figure 2B
Pronase E (0.1 %) - + + + + +
DISS (μM) 0 0 1 3 10 30
150 kDa
100 kDa
SIRT1
50 kDa
40 kDa
β-actin
The third result of Figure 2B
Pronase E (0.1 %) - + + + + +
DISS (μM) 0 0 1 3 10 30
150 kDa
100 kDa
SIRT1
50 kDa
40 kDa
β-actin

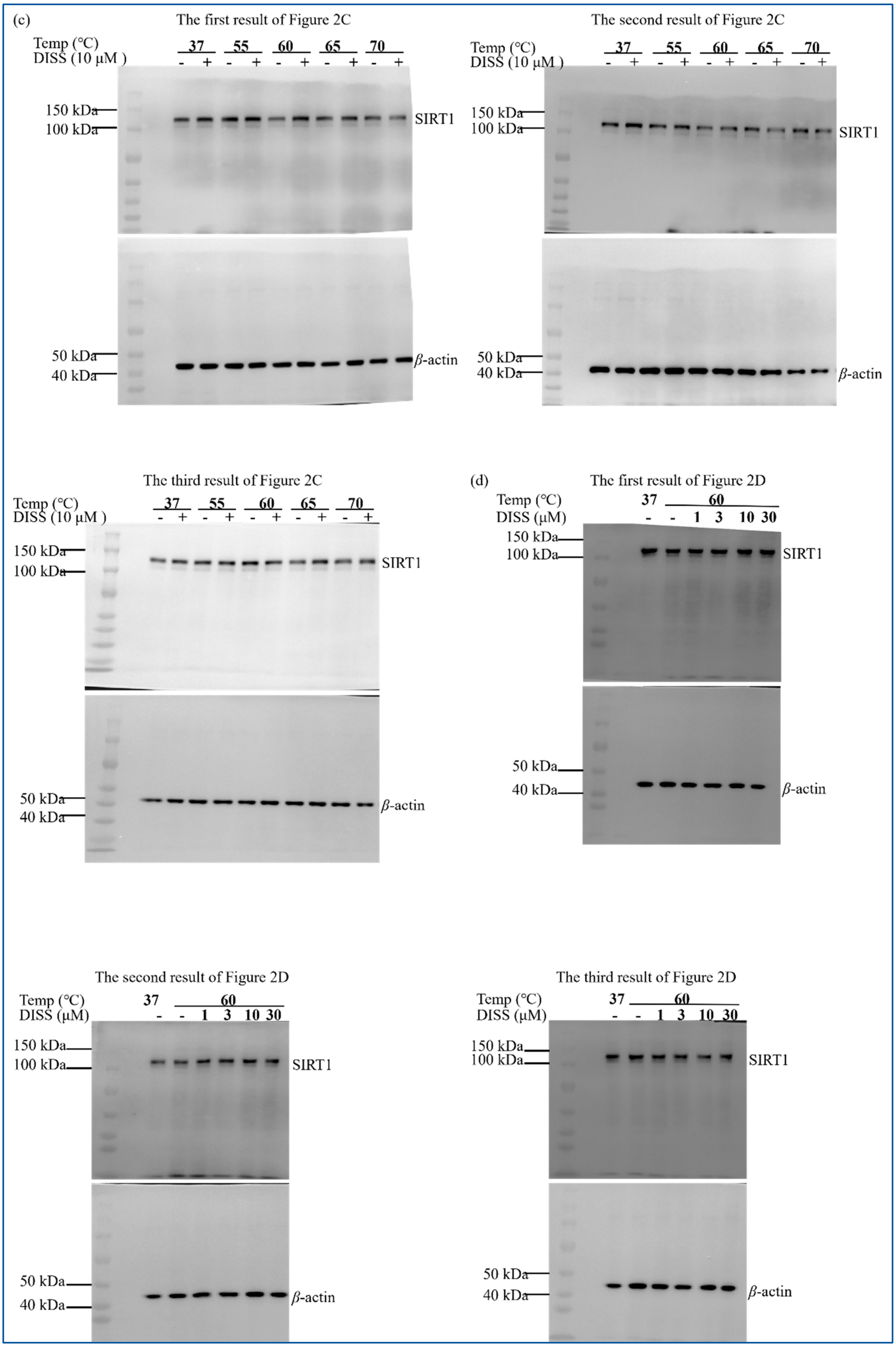
(c)
The first result of Figure 2C
Temp (°C) 37 55 60 65 70
DISS (10 μM) - + - + - + - + - +
150 kDa
100 kDa
SIRT1
50 kDa
40 kDa
β-actin
The second result of Figure 2C
Temp (°C) 37 55 60 65 70
DISS (10 μM) - + - + - + - + - +
150 kDa
100 kDa
SIRT1
50 kDa
40 kDa
β-actin
The third result of Figure 2C
Temp (°C) 37 55 60 65 70
DISS (10 μM) - + - + - + - + - +
150 kDa
100 kDa
SIRT1
50 kDa
40 kDa
β-actin
(d)
The first result of Figure 2D
Temp (°C) 37 60
DISS (μM) - - 1 3 10 30
150 kDa
100 kDa
SIRT1
50 kDa
40 kDa
β-actin
The second result of Figure 2D
Temp (°C) 37 60
DISS (μM) - - 1 3 10 30
150 kDa
100 kDa
SIRT1
50 kDa
40 kDa
β-actin
The third result of Figure 2D
Temp (°C) 37 60
DISS (μM) - - 1 3 10 30
150 kDa
100 kDa
SIRT1
50 kDa
40 kDa
β-actin

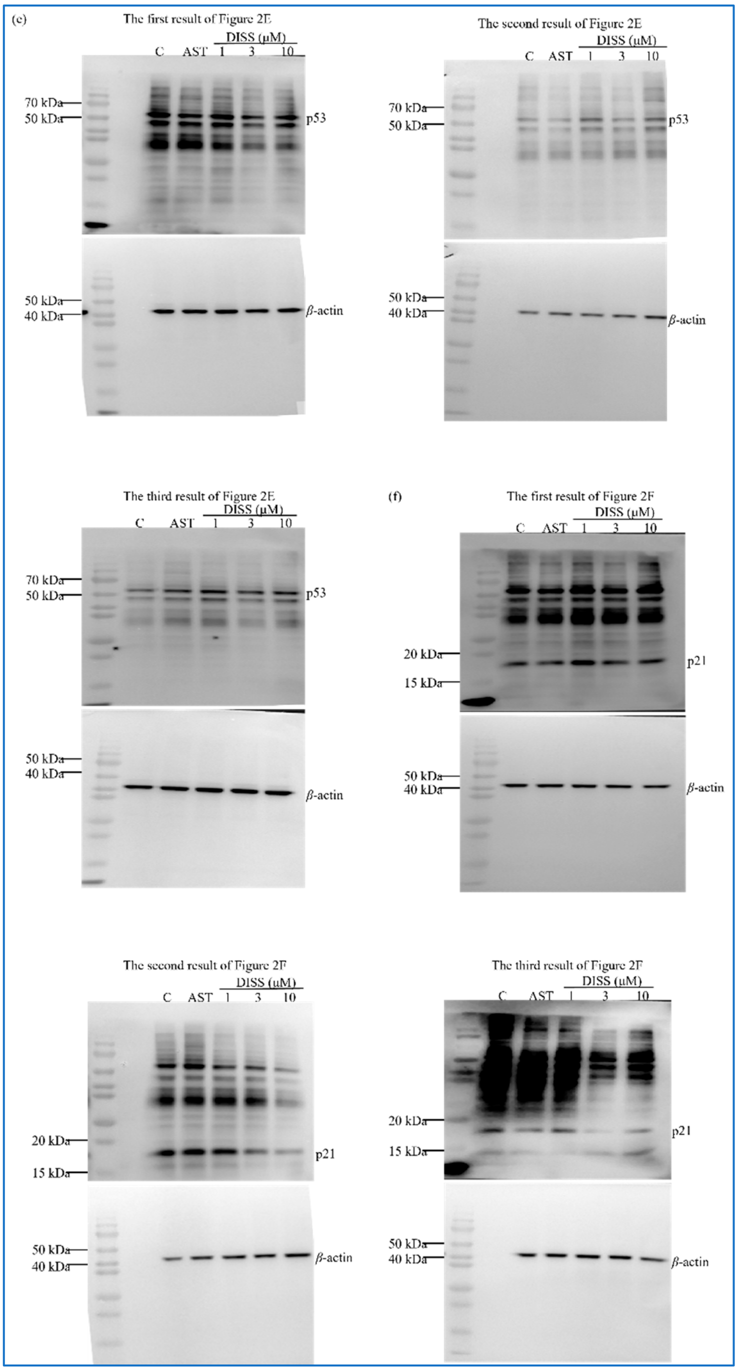

Supplementary Figure S3. Original data of western blot analysis of the effect of DISS on SIRT1, p53, p21 and *β*-actin in Figure 2. (a) Original data of western blot analysis of the effect of AST and doses of DISS on SIRT1 and *β*-actin in Figure 2A. (b) Original data of western blot analysis of the effect of 0.1% pronase E and doses of DISS on SIRT1 and *β*-actin in Figure 2B. (c) Original data of western blot analysis of the effect of 10 μM DISS and different temperatures on SIRT1 and *β*-actin in Figure 2C. (d) Original data of western blot analysis of the effect of doses of DISS and heating at 60 °C on SIRT1 and *β*-actin in Figure 2D. (e) Original data of western blot analysis of the effect of AST and doses of DISS on p53 and *β*-actin in Figure 2E. (f) Original data of western blot analysis of the effect of AST and doses of DISS on p21 and *β*-actin in Figure 2F.

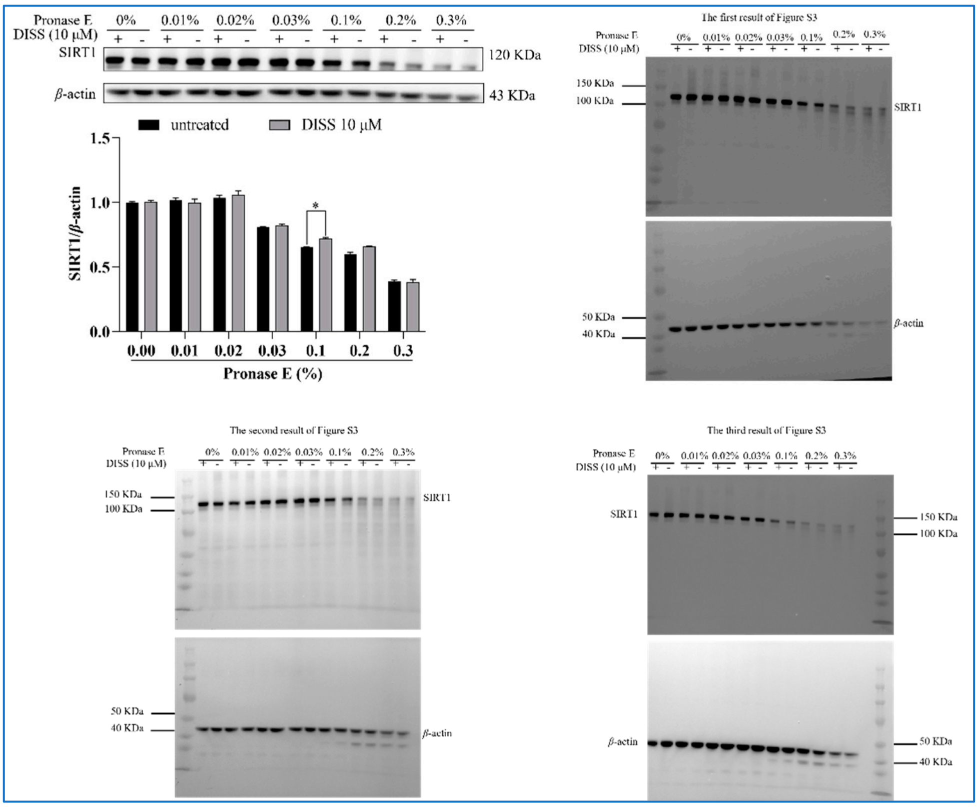

Supplementary Figure S4. The western blot stripes, digitized results and original data of western blot analysis of the effect of 10 μM DISS and different concentrations of pronase E on SIRT1 and β-actin.

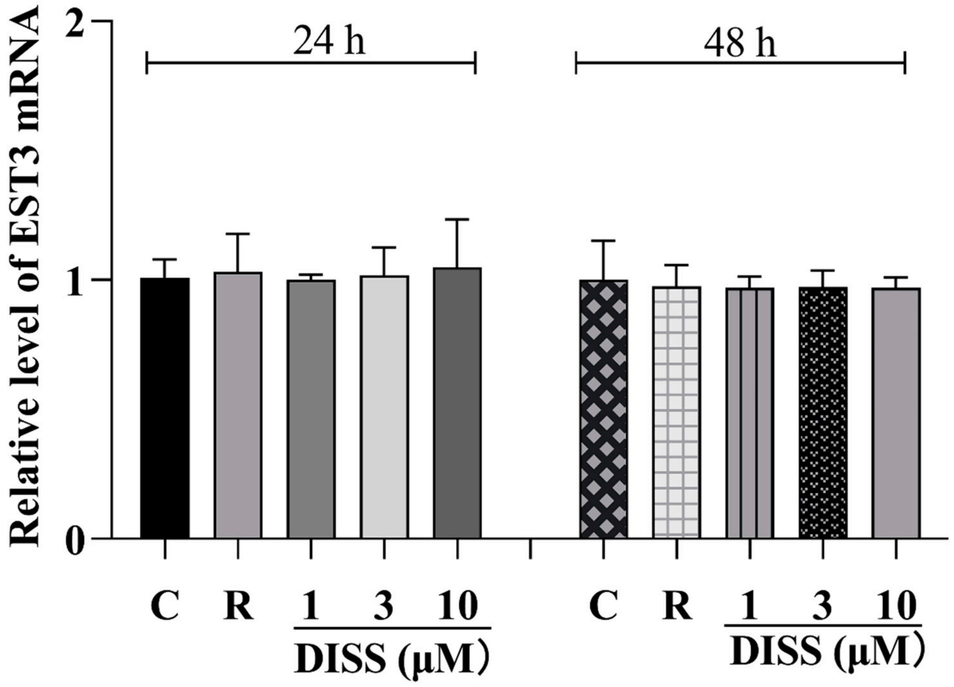

Supplementary Figure S5. Effect of DISS treatment on the expression of telomerase-related genes EST3 for 24h or 48h.

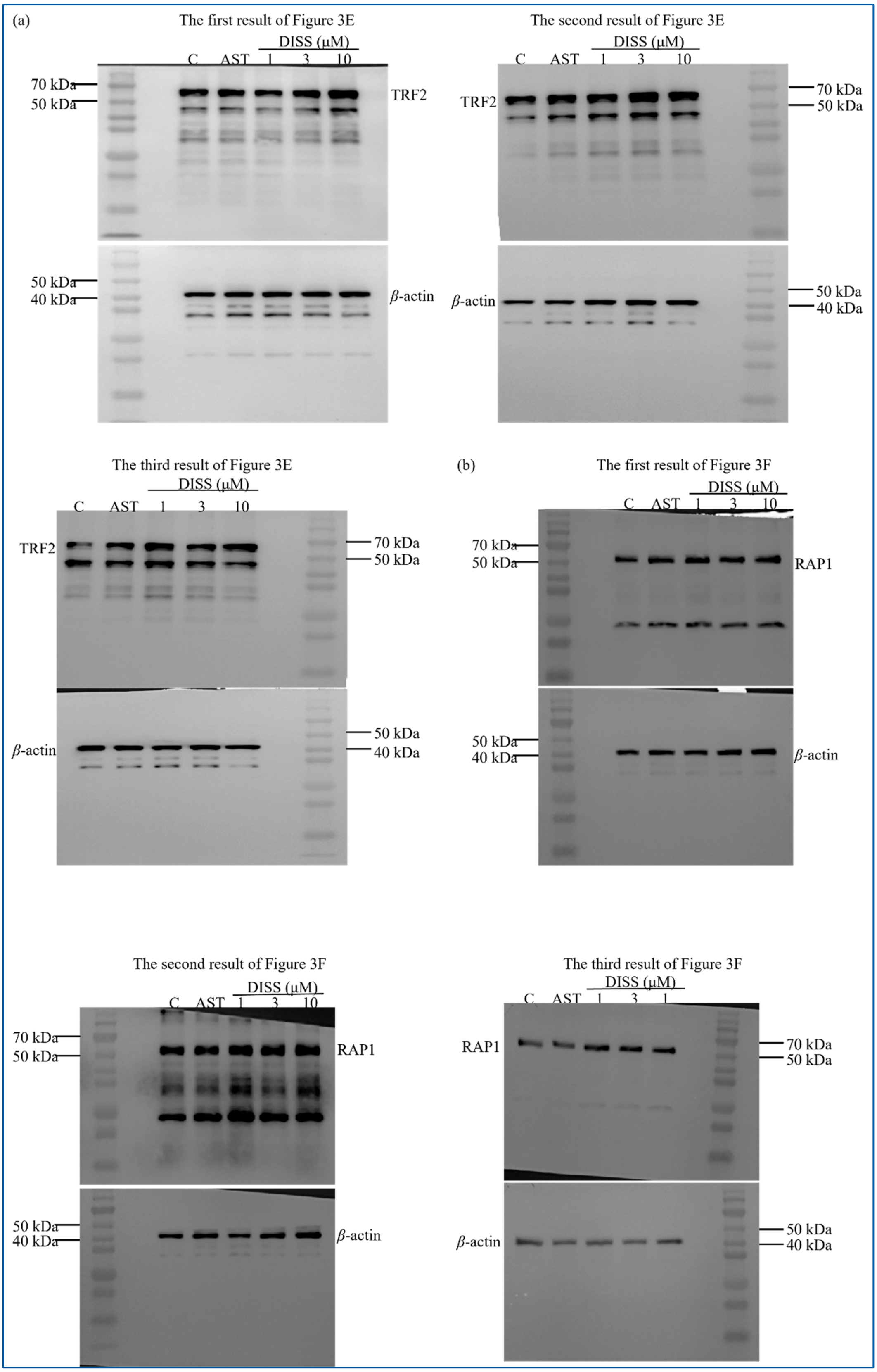

Supplementary Figure S6. Original data of western blot analysis of the effect of DISS on TRF2, RAP1 and *β*-actin in Figure 3E and Figure 3F. (a) Original data of western blot analysis of the effect of AST and doses of DISS on TRF2 and *β*-actin in Figure 3E. (b) Original data of western blot analysis of the effect of AST and doses of DISS on RAP1 and *β*-actin in Figure 3F.

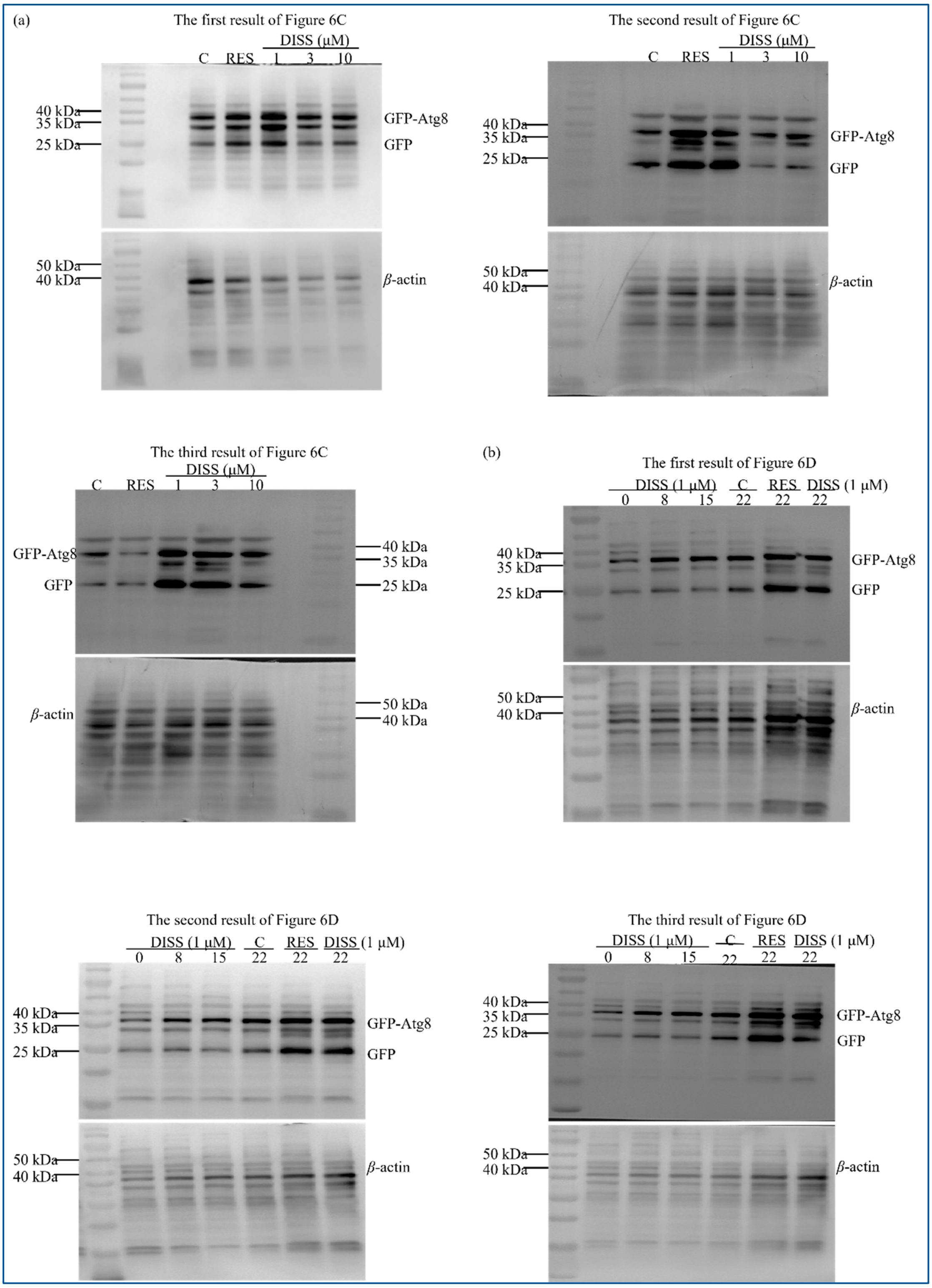

Supplementary Figure S7. Original data of western blot analysis of the effect of DISS on GFP and *β*-actin in Figures 6C and 6D. (a) Original data of western blot analysis of the effect of RES and doses of DISS on GFP in Figure 6C. (b) Original data of western blot analysis of the effect of RES and 1 μM DISS on GFP at different times in Figure 6D.

References

[1] Chen, S.Q.; Li, Y.J.; Wu, E.C.; Li, Q.; Xiang, L.; Qi, J.H. Arctigenin from Fructus arctii Exhibits Antiaging Effects via Autophagy Induction, Antioxidative Stress, and Increase in Telomerase Activity in Yeast. *Antioxidants* **2024**, 13, 684, doi:10.3390/antiox13060684.

[2] Shan, J.H.; Mo, J.X.; An, C.Y.; Xiang, L.; Qi, J.H. β-Cyclocitral from Lavandula angustifolia Mill. exerts anti-Aging effects on yeasts and mammalian cells via telomere protection, antioxidative stress, and autophagy activation. *Antioxidants* **2024**, 13, 715, doi:10.3390/antiox13060715.

[3] Qi, H.Y.; Su, F.Y.; Chen, Y.J.; Cheng Y.Q.; Liu A.J. The Antiaging Activity and Cerebral Protection of Rapamycin at Micro-doses. *CNS Neuroscience & Therapeutics* **2014**, 20, 991-998, doi:10.1111/cns.12338.

[4] Dikicioglu, D.; Dereli Eke, E.; Eraslan, S.; Oliver, S.G. *Saccharomyces cerevisiae* adapted to grow in the presence of low-dose rapamycin exhibit altered amino acid metabolism. *Cell Commun Signal* **2018**, 16, doi:10.1186/s12964-018-0298-y.

[5] Foster, K.G.; Fingar, D.C. Mammalian Target of Rapamycin (mTOR): Conducting the Cellular Signaling Symphony. *Journal of Biological Chemistry* **2010**, 285, 14071 – 14077, doi:10.1074/jbc.r109.094003.

[6] Ma, R.Y.; Yu, R.M.; Peng, Y.; Yi, H.F.; Wang, Y.C.; Cheng, T.F; Shi, B.Q.; Yang, G.; Lai, W.M.; Wu, X.S. Resveratrol induces AMPK and mTOR signaling inhibition-mediated autophagy and apoptosis in multiple myeloma cells. *Acta Biochimica et Biophysica Sinica* **2021**, 53, 775 – 783, doi:10.1093/abbs/gmab042.
